# Exploring Nile Red and machine learning for microplastics detection in *Tridacna maxima*

**DOI:** 10.1371/journal.pone.0357014

**Published:** 2026-09-16

**Authors:** Irène Godéré, Taiamiti Edmunds, Nabila Gaertner-Mazouni, Fiona Gimenez, Pascal Wong-Wah-Chung, Stéphanie Lebarillier, Magalie Baudrimont, Chloé Pupier, Nicolas Maihota, Jean-Claude Gaertner

**Affiliations:** 1 Université de Polynésie Française, UMR SECOPOL, Tahiti, Polynésie Française; 2 AI Freelancer Papeete, Tahiti, Polynésie Française; 3 Aix-Marseille Université, LCE, Aix-en-Provence, France; 4 Université de Bordeaux, CNRS, UMR EPOC 5805, Pessac, France; 5 Institut de Recherche pour le Développement, UMR SECOPOL 241, Tahiti, Polynésie Française; VIT University, INDIA

## Abstract

Small Island Developing States (SIDS) face unique challenges for microplastics (MPs) monitoring due to limited infrastructure and resources. In this context, we propose and test innovative approaches toward a standardized, low-cost methodology for quantifying MPs in SIDS. We evaluate the giant clam *T. maxima* as a bio-integrator, combining Nile red (NR) fluorescence staining with automated machine-learning detection. We optimized a digestion protocol using KOH and HNO_3_ for *T. maxima* viscera, and developed a DAPI-guided multi-spectra composite imaging approach based on triband fluorescence (DAPI, FITC, TRITC), to enhance polymer detection while reducing blooming artifacts. A semi-automated annotation pipeline using Labkit interactive segmentation with CLIP/UMAP clustering efficiently generated training data from 6711 fluorescence images. A U-Net model was trained on composite images to segment fluorescent particles. The workflow was applied to giant clams from three French Polynesian islands (Makemo, Hao, Tubuai), and NR-based estimates were validated against µFTIR spectroscopy. The model achieved F1-scores of 0.741 for giant clam samples and 0.657 for controls, comparable to human annotation (F1 = 0.680). MPs were detected across all islands, with highest concentrations in gills (16.9–52.7 particles·g^−1^ wet weight) compared to viscera (2.5–11.0 particles·g^−1^ ww). µFTIR validation revealed that NR overestimates MP counts (µFTIR: 0.80 ± 0.16 particles·g^−1^ ww at Tubuai), primarily due to false positives from proteins, cellulose, and stearates. In Tubuai, polyamide (28.9%), PVC (12.6%), and polystyrene (10.7%) were the dominant polymers, suggesting contributions from fishing gear, agriculture, and household waste. While NR-based quantification overestimates absolute MP counts, the automated pipeline demonstrates potential for high-throughput image processing, reproducible sample analysis, and methodological standardization. This workflow represents a first step toward scalable, low-cost approaches for MPs monitoring in insular systems, highlighting areas for further calibration and optimization. Future work should refine fluorescence thresholds and expand validation across species and locations.

## 1. Introduction

Microplastics (MPs) are now recognized as pervasive contaminants across marine ecosystems worldwide, with documented occurrences in coastal sediments, water columns, coral reef systems, and numerous marine organisms [1–3]. In French Polynesia (FP), occasional studies have reported the presence of MPs in pearl oysters [4], reef fishes [5,6] and beach sand [7], yet comprehensive monitoring remains limited. Small Island Developing States (SIDS), including FP, face unique challenges for MP monitoring due to restricted laboratory infrastructure, limited access to advanced analytical equipment, and the need for low-cost yet reliable methods suitable for remote settings.

In this context, biological sentinels represent a feasible approach for MPs monitoring. Among them, bivalve mollusks are widely used as bio-integrators of environmental contamination in temperate environments due to their filtration capacity and ecological relevance [8,9]. The giant clam *Tridacna maxima* is particularly promising for tropical environments, as it has been shown to ingest MPs [10,11] and reflect environmental contamination [12]. However, the absence of standardized sample processing protocols, especially for *T. maxima* with its high biomass and complex tissues, limits their use as bio-integrators for large scale MPs monitoring [9]. Standard analytical approaches such as micro-Fourier Transform Infrared Spectroscopy (µ-FTIR) and Raman spectroscopy provide reliable polymer identification and allow discrimination between synthetic and non-synthetic particles [13,14], but these methods remain expensive, time-consuming and difficult to deploy at scale in remote islands. As a result, low-cost fluorescence-based screening methods, especially Nile Red (NR) staining [15–19], have gained attention. NR selectively binds to hydrophobic materials, such as plastics, enabling rapid visualization of MPs under fluorescence microscopy [19]. However, NR workflows face several limitations, with the interference from natural organic matter, variability in fluorescence intensity across particle types, and the risk of false positives (e.g., lipids, cellulose, non-synthetic fibers) [16–18]. Traditional particle detection approaches based on intensity thresholding (e.g., Image J, MP-VAT, and MP-VAT 2.0 tools) [15,18], often fail when applied to environmental samples rich in biological debris, variable illumination, or heterogeneous backgrounds. Recent deep-learning approaches, such as MP-Net [20], improve segmentation MPs accuracy in NR-stained microscopy images, but these models were developed and evaluated primarily on curated datasets of spiked synthetic particles or clean samples, which may not fully capture the heterogeneity of real-world environmental matrices. Current NR-based machine learning workflows also rely on fully manual annotation of ground-truth masks, which is prohibitively time-consuming for large-scale ecological studies. Finally, single-wavelength fluorescence imaging limits polymer detection, as different polymer types exhibit optimal fluorescence at different excitation wavelengths [16–18]. Multi-channel imaging increases coverage but requires careful methodology to manage differential exposure levels, background interference, and blooming artifacts, where light spreads from bright particles into adjacent areas due to the point spread function (PSF). This effect can distort particle size and lead to overestimation of particle mass, introducing systematic bias in contamination assessments.

To address these challenges, this study specifically aimed to: (i) optimize digestion and purification protocols to efficiently isolate MPs from *T. maxima*, (ii) develop a standardized NR fluorescence workflow coupled with machine-learning-based segmentation, (iii) benchmark NR-derived estimates against µFTIR polymer identification, and (iv) describe island-scale contamination patterns relevant to resource-limited SIDS monitoring programs.

To achieve these goals, we implemented an integrated, end‑to‑end methodological framework combining laboratory processing and computational analysis. MPs extraction protocols were optimized to process *T. maxima* tissues while preserving particle integrity, with the goal of obtaining samples suitable for fluorescence-based detection. This was followed by a multi-spectral fluorescence imaging strategy based on triband acquisition (DAPI–FITC–TRITC), with deliberate emphasis on the DAPI channel to enhance polymer detection, reduce blooming artifacts, and improve visual interpretability for human annotation. We then introduced a scalable semi-automatic annotation pipeline that integrates Labkit’s interactive segmentation tools with batch processing and cluster-based correction using FiftyOne, coupled with a lightweight convolutional neural network capable of efficiently segmenting complex environmental datasets. Finally, NR-based quantification was validated against µFTIR polymer identification on environmental particles, ensuring accurate discrimination between synthetic and non-synthetic particles and allowing confidence in the method’s ecological relevance.

Overall, this study establishes a reproducible, low‑cost, and scalable workflow – from digestion to imaging, machine learning, and spectroscopic validation – that enhances MPs monitoring capacity in remote islands and provides new methodological insights for MP quantification in complex biological matrices.

## 2. Materials and methods

### 2.1. Study sites and sampling

Giant clams were collected on three islands in FP, selected for their contrasting levels of anthropogenic activities.

Makemo (16°36′S, 143°41′W) located in the Tuamotu archipelago, is an atoll with low human influence, dominated by coconut production (197 tons in 2017) and small-scale pearl farming (17 ha) [21], with a low population density (15 inhabitants.km^−2^ [22]). The lagoon of 603 km^2^ has a relatively short water residence time, estimated at 15 days [23].

Hao (18°12′S, 140°55′W), also situated in the Tuamotu archipelago, is a former military site that hosted the Centre d’Expérimentation du Pacifique (CEP) during nuclear tests from 1964 to 2000. It has a population density of 22 inhabitants.km^−2^ [22] and a lagoon (720 km^2^) with a water residence time of 90 days [23].

Tubuai (23°23′S, 149°28′W), in the Australes archipelago, is an agricultural island supplying nearly 70% of the agricultural produce value of the archipelago [24] and is a significant source of giant clams (40.6 tons collected annually for consumption [25]). Its lagoon (85 km^2^) is continuously renewed through two reefs [26], and the island has a population density of 40 inhabitants.km^−2^ [22].

On each island, three stations were sampled to account for local variability: S1 near the village, S2 close to a dominant anthropogenic activity (*e.g.,* coconut plantation in Makemo, military base in Hao, and agriculture in Tubuai), and S3 in a relatively undisturbed area, primarily influenced by oceanic inputs. GPS coordinates for all sampling stations are provided in Table 1.

**Table 1:** GPS coordinates of sampling stations.

Island	Station	Latitude (°S)	Longitude (°W)
Makemo	S1	16°39′00.7″S	143°34′53.2″W
Makemo	S2	16°40′33.3″S	143°37′41.5″W
Makemo	S3	16°28′05.1″S	143°56′47.0″W
Hao	S1	18°09′59.3″S	141°00′36.9″W
Hao	S2	18°21′11.3″S	140°47′01.1″W
Hao	S3	18°04′48.8″S	141°00′37.8″W
Tubuai	S1	23°20′09.9″S	149°28′23.2″W
Tubuai	S2	23°21′49.1″S	149°32′12.9″W
Tubuai	S3	23°24′51.5″S	149°29′45.6″W

Five giant clams were randomly collected at each station from June 2021 to February 2023, and frozen before analysis in the laboratory. We also collected additional samples (n = 13) in Tubuai for further comparative analysis (µFTIR). The additional sampling effort was carried out on this island because it constitutes a significant source of giant clams for human consumption in FP [25]. In total, 45 giant clams were analyzed by NR fluorescence microscopy (15 per island, 5 per station), and 13 were analyzed by µFTIR (Tubuai only). Shell length (mean ± SE) averaged 159.6 ± 3.4 mm in Tubuai, 149.7 ± 4.4 mm in Hao, and 132.5 ± 4.2 mm in Makemo, while total wet masses reached 123.1 ± 7.6 g, 90.0 ± 8.1 g, and 71.1 ± 7.1 g, respectively (S1 Table).

No collection permits or ethics approval were required for this study. All sampled giant clams exceeded the minimum shell length of 12 cm established by French Polynesian regulations [27]. Research involving non-cephalopod invertebrates is not subject to ethical review under French and European regulations (Directive 2010/63/EU, transposed into French law via Décret n° 2013−118). Informed consent was not applicable.

### 2.2. Optimizing digestion protocols for MPs extraction from giant clams

In the laboratory, giant clams were dissected in order to collect the gills and *viscera* (including the Gonad and Digestive System (GDS) and kidneys), which are known organs of interest in bivalves in particular [28]. On average, 4.03 ± 0.17 g ww of gill tissue and 36.50 ± 2.20 g ww of viscera per individual were processed for digestion (see S1 Table for individual values). Tubuai giant clams had the largest gills (4.60 ± 0.29 g ww) and viscera (45.8 ± 3.9 g ww), followed by those from Hao and Makemo (S1 Fig). The initial digestion consisted of treating gills and *viscera* with 10% potassium hydroxide (40°C, 120 rpm agitation, 1:10 volume ratio) for 48h [29,30]. For NR analysis, the digestates were then sieved to 20 µm on a stainless-steel sieve, and particles were resuspended before filtration on pyrolyzed GF/C filter. As *viscera* showed significant organic residues despite the KOH digestion, six alternative protocols from the literature were tested to optimize digestion efficiency. First, digestion time was extended: 10% KOH at 40°C for 24 h followed by 7 days at room temperature [11]. Second, the effects of microwave and ultrasound assistance were assessed: 30 min sonication immediately after KOH addition, followed by 72 h at 40°C, and a final microwave treatment (500 W, 3 min) [12,31]. Third, a double digestion approach was evaluated after initial KOH treatment, using either 30% hydrogen peroxide (H_2_O_2_, 250 mL per sample, 40°C for 2 h [4]) or 20% concentrated nitric acid (HNO_3_, 40 mL added on GF/C filters, room temperature, 1 h [32]).

The most effective digestion protocol was subsequently evaluated for its impact on polymer integrity. Fragments (<5 mm; n = 3 per polymer type) from six commonly encountered polymers (PE, PP, PVC, PA, PET, and a PE–PP copolymer representative of ropes used in pearl farming) were prepared and spiked into giant clam viscera tissue. Fragments were recovered at each stage of the procedure (before digestion, after the first digestion step, and after the second digestion step), photographed, and measured (length and width) to assess potential physical alterations. Representative particles were also analyzed by ATR-FTIR spectroscopy to detect changes in chemical structure or spectral features induced by digestion. This approach allowed evaluation of polymer integrity and estimation of recovery rates under the applied digestion protocol.

For µ-FTIR analyses of field samples, additional samples from Tubuai (n = 13) were digested using the optimized protocol, then particles were collected on a 20 µm stainless-steel sieve, before being re-suspended and filtered through stainless-steel filters with different mesh sizes (220 µm, 110 µm, 58 µm, 25 µm, 13 µm) before analysis.

**Fig 1:**
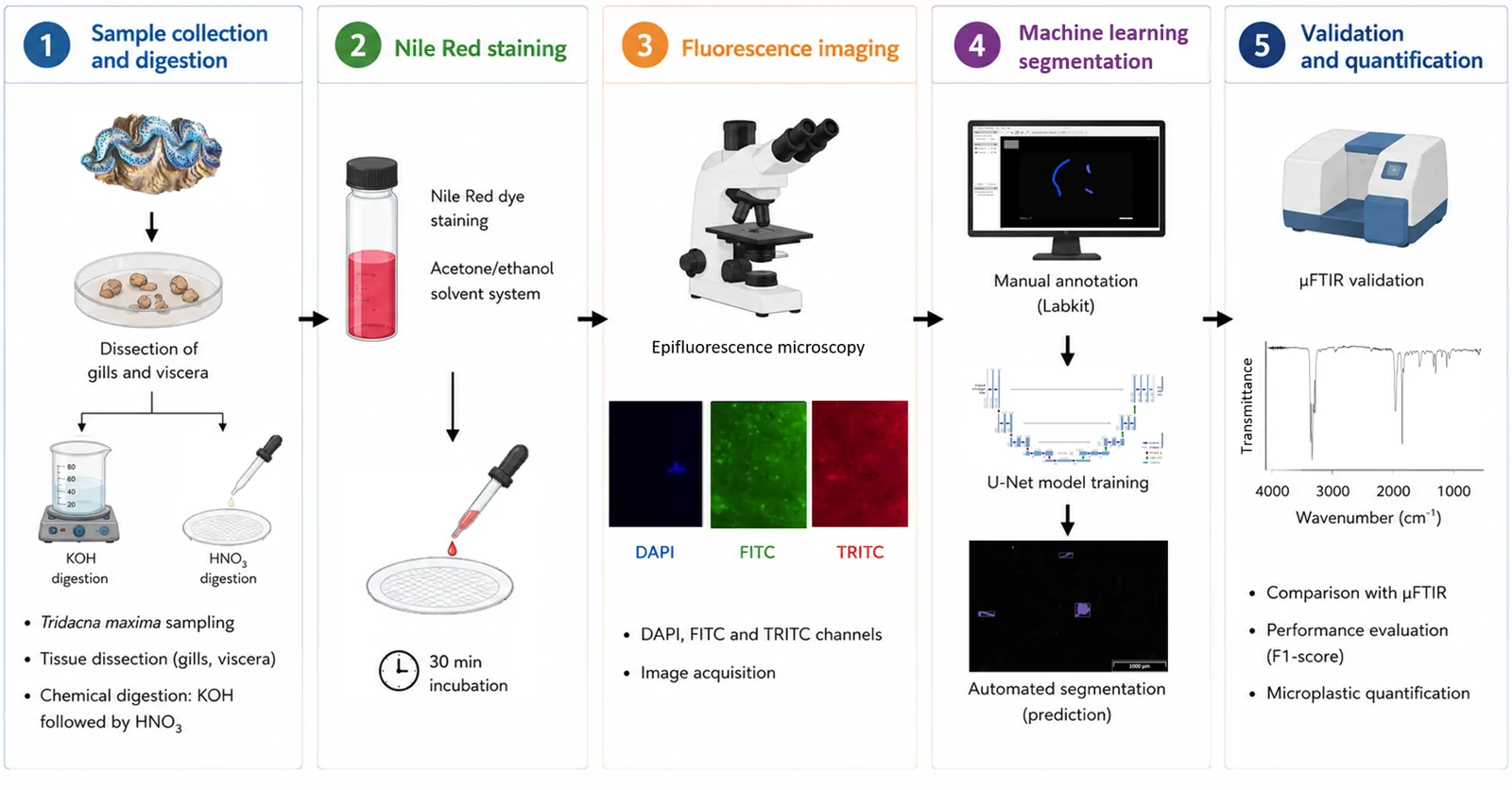
Schematic overview of the analytical workflow used for MPs detection in *Tridacna maxima.*

A schematic overview of the analytical workflow, from sample digestion to validation (digestion, staining, imaging, segmentation, and µFTIR confirmation), is provided in Fig 1 to facilitate methodological clarity and reproducibility.

To reduce contamination by airborne particles, all solutions (i.e., Milli-Q water, 10% KOH, 30% H_2_O_2_, 20% HNO_3_) were filtered through glass microfiber filters GF/F (Whatman®). Glass and stainless-steel equipment were used and rinsed with filtered water, and jars were immediately covered with aluminum foil. To minimize contamination within the laboratory, all samples were handled in a fume hood and operators wore cotton lab coats. In addition, to account for possible contamination during the sample handling process, controls (referred to as “CBENI”) containing filtered water (GF/F) were prepared and left open inside the fume hood each time the glass jars containing the samples were opened. These controls were analyzed in the same way as the giant clam samples in order to quantify possible external contamination.

### 2.3. Nile red staining and images acquisition

#### 2.3.1. Nile Red staining.

The particles extracted on GF/C filters were then stained with NR using the method of Konde et al. [33]. A volume of 0.5 mL of a 20 µg.mL^-1^ solution of NR (in equal proportions of acetone and ethanol) was added to the GF/C filters and heated at 50°C for 10 minutes.

#### 2.3.2. Image acquisition.

The GF/C filters were then observed under an epifluorescence microscope (Olympus IX73) equipped with a triband fluorescence filter: DAPI (Ex 355–405 nm), FITC (Ex 453–483 nm), TRITC (Ex 540–575 nm). Because certain fluorescence filters exhibit poor detection of certain plastic polymers due to their polarity (absence or weak fluorescence), the triband filter was used to detect a wide range of plastic polymers by combining the three most commonly used filters in plastics studies [16,17].

The entire sample filter was scanned under the microscope. Areas where fluorescent particles were visible were designated as regions of interest (ROI), and captured under the triband filter (TRI). For each ROI, captures were also made under two individual polarized filters (DAPI: dapi filter and FITC: fitc filter; which are initially contained in the triband, and appeared to be underestimated). Captures were also made under bright field conditions (NAT). Particular care was taken to avoid superimposing particles on two different images, and to prevent cutting off particles. These marginal cases were rare, as MPs were generally isolated. The four images were captured with an Olympus® DP74 color camera, at a resolution of 1920 x 1200 pixels, with exposure times tailored to each filter (triband ≈ 6 ms; DAPI ≈ 50 ms; FITC ≈ 200 ms; bright field ≈ 1 ms), and an objective magnification varying from x4 to x10 depending on particle size. Exposure times for the various filters were set as values that would best avoid under- or over-exposed areas of the image, since a non-uniform dispersion of the dye related to particle charge was observed in a few areas of some samples.

**Fig 2:**
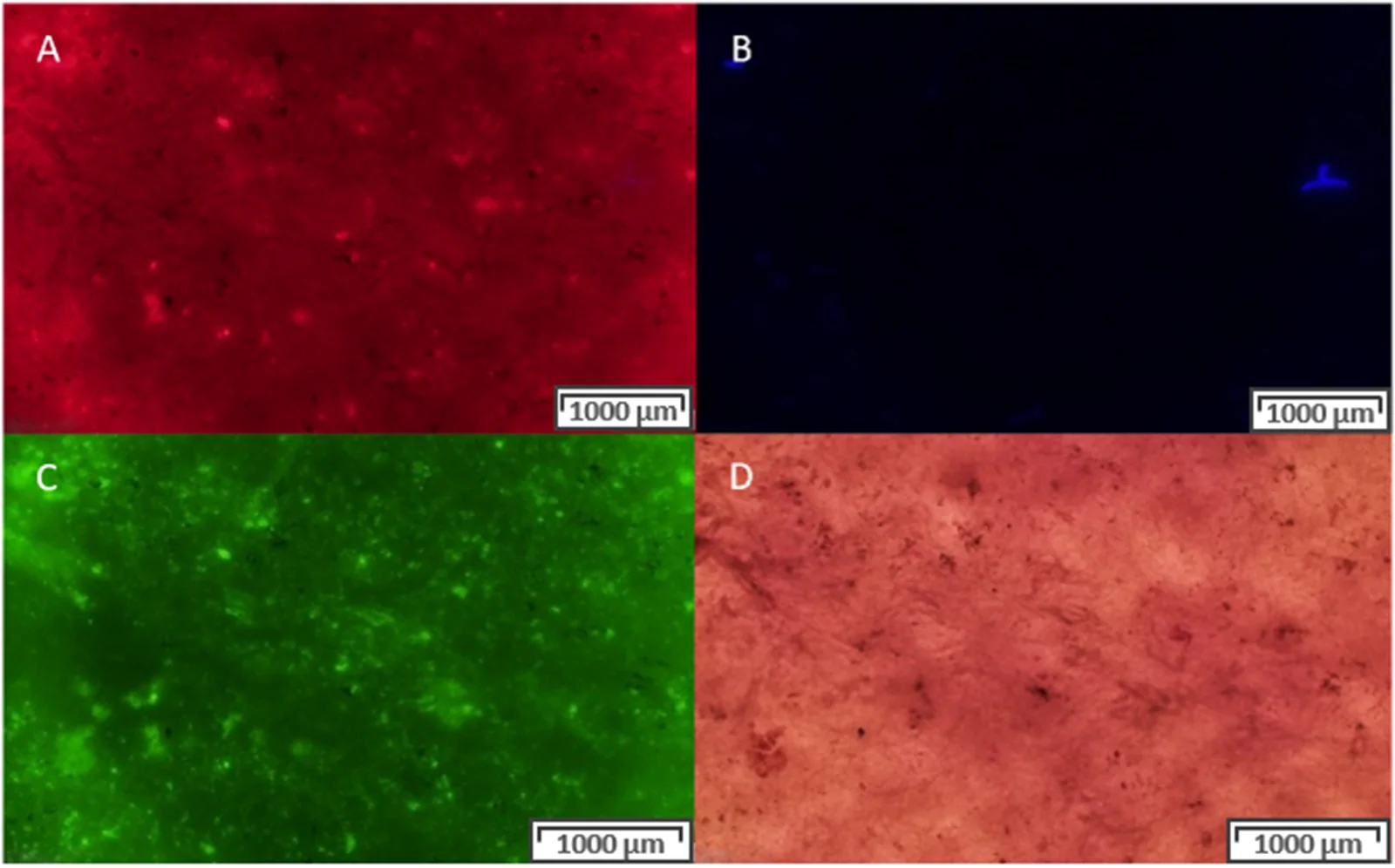
Fluorescence responses of GF/C filters under TRITC (A), DAPI (B), FITC (C), and NAT (D) channels (4x objective).

Certain particles were visible in the four filters while some were visible in a single filter (Fig 2). TRI and DAPI were the most effective filters to highlight MPs without emphasizing other particles related to organic matter. Both TRI and DAPI were retained for the rest of the data processing pipeline. Future work could leverage both FITC and NAT filters but they were discarded in this study.

#### 2.3.3. Data statistics.

Across the 45 giant clams included in this study (15 individuals from each of the three islands), an average of approximately four GF/C filters were analyzed per individual, although a larger number were required for some samples, particularly for the largest individuals. On average, 133 ± 6 ROIs (SE) were acquired per individual, with values ranging from 110 to 172 ROIs depending on the sample (Table 2).

**Table 2:** Overview of biological and imaging dataset structure: number of islands and *Tridacna maxima* individuals sampled, mean number of GF/C filters per clam, and mean number of ROIs analyzed per sample. Data are presented as mean ± SE (min–max).

Number of islands	Number of giant clams by island (sample)	Mean number of GF/C filters by individual (subsample)	Mean number of ROI by sample
3	15	3.7 ± 0.1 (2-11)	133 6 (110-172)

### 2.4. Multi-spectra image composite

#### 2.4.1. Filter selection and preliminary analysis.

To determine the optimal fluorescence imaging strategy, preliminary analyses were conducted comparing MPs detection across different fluorescence filters. Three filter configurations were evaluated: DAPI (355–405 nm), FITC (453–483 nm) and TRI (a multi-wavelength filter combining DAPI, FITC, and TRITC (540–575 nm)) [16,17]. Each filter exhibited distinct advantages and limitations as shown in Fig 1. DAPI provides a strong fluorescence signal for detecting MPs, but limited coverage of certain polymer types due to a narrow excitation range. FITC allowed detection of specific polymers but was highly sensitive to organic residuals, generating substantial background noise. And TRI combined detection capability across multiple wavelengths commonly used in the literature for MPs, but exhibited low contrast, making human identification of MPs challenging when used alone. Based on this preliminary analysis, we developed a composite image approach combining DAPI (single wavelength) and TRI (multi-wavelength) channels. While the TRI filter inherently includes FITC wavelengths, the composite strategy mitigates FITC-associated noise by leveraging differential spectral responses between the two channels.

#### 2.4.2. Three-stage composite image processing.

The multi-spectra composite serves three critical functions. It enables detection of diverse polymer types within a single image through complementary wavelength coverage, enhances visual discrimination of MPs for human annotation through color-coded representation, and mitigates blooming artifacts caused by PSF effects in overexposed structures.

Composite images were generated through a three-stage process. First, individual illumination correction was applied: uneven illumination was corrected within each channel independently to normalize background intensity across the field of view. This addresses vignetting and uneven excitation common in wide-field fluorescence microscopy, using a background estimation approach based on local intensity smoothing [34]. Second, illumination alignment was performed: since DAPI typically exhibited higher exposure than the corresponding TRI channel, histogram-matching was used to redistribute DAPI intensities to match the mean and standard deviation of the TRI channel, equalizing exposure levels while preserving relative contrast [35].

**Fig 3:**
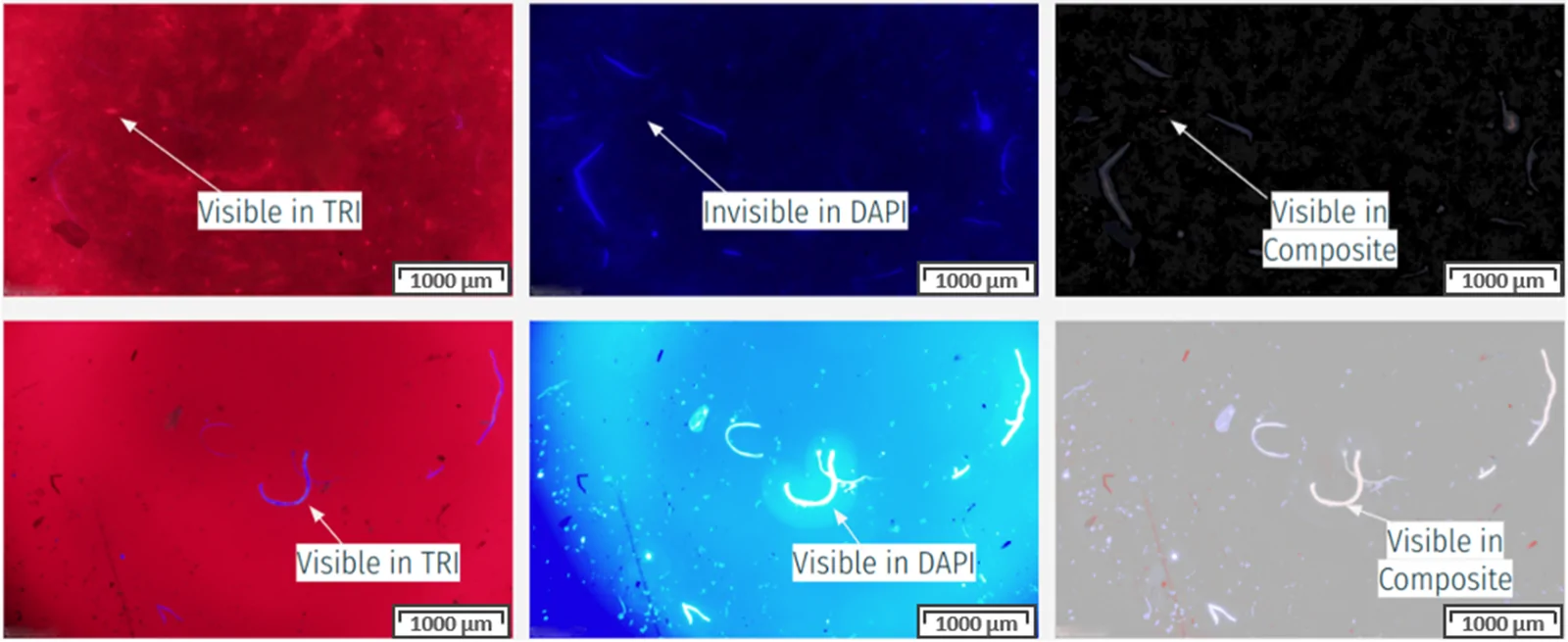
Example of fluorescence images from the same ROI: TRITC (A), DAPI (B), and composite (C) (4x objective). The composite employs a color-coded scheme where red indicates TRI-dominant fluorescence, blue indicates DAPI-dominant fluorescence, and white indicates strong signal in both channels. This color scheme enables immediate visual discrimination of polymer specific fluorescence patterns and facilitates human annotation while reducing blooming artifacts through differential exposure between channels.

**Fig 4:**
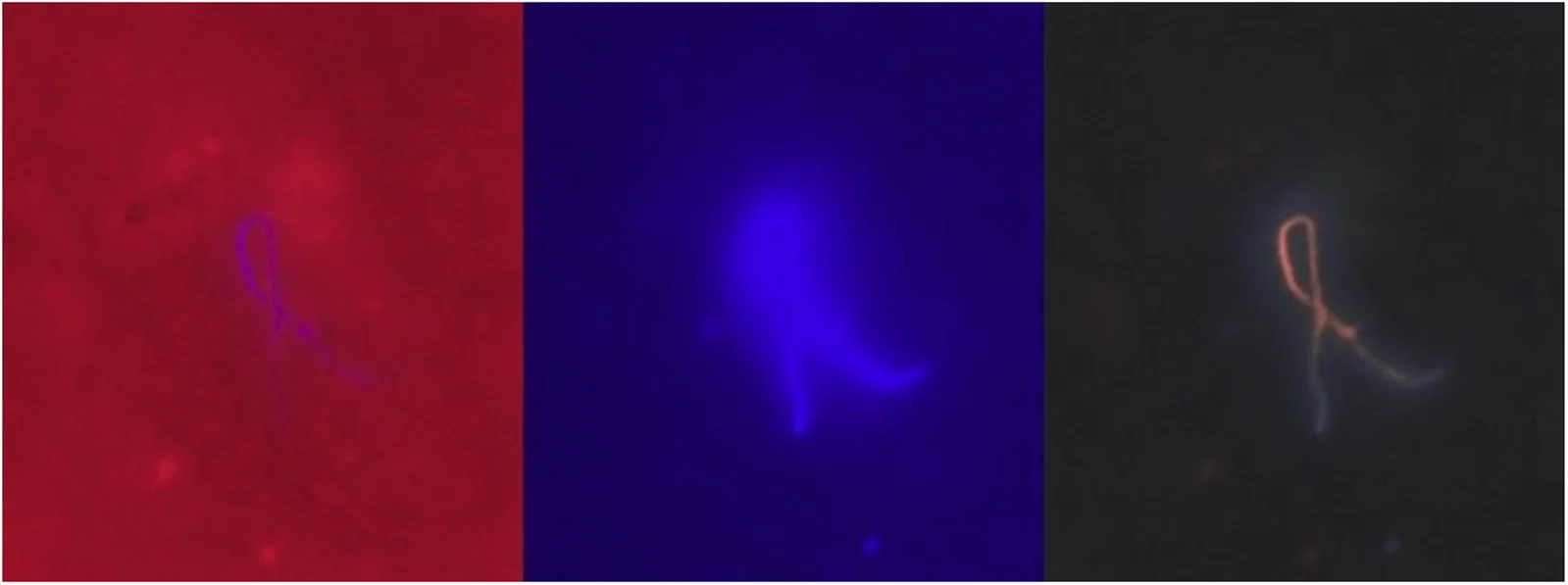
Example of fluorescence images from the same ROI: TRI (A), DAPI (B), and composite (C) (4x objective). Blooming is observed in DAPI and low contrast is observed in TRI. A sharp contour is observed in image composite.

Third, a color-coded overlay was applied to create interpretable outputs, where red structures indicate strong TRI fluorescence with weak or absent DAPI signal, blue structures correspond to strong DAPI fluorescence with weak or absent TRI signal, white structures show strong fluorescence in both channels, and black or dark represents background or weak signal in both channels. This color scheme enables immediate visual identification of polymer-specific fluorescence patterns while reducing blooming artifacts as shown in Fig 3 and Fig 4. When a bright particle is overexposed in one channel (e.g., DAPI), the PSF spreads light into neighboring pixels, artificially enlarging the particle’s apparent size. By combining with the TRI channel, the composite image provides a more accurate representation of particle boundaries, constraining the blooming-induced area inflation that would otherwise affect mass estimates derived from projected area measurements.

### 2.5. Model for automatic detection and quantification of MPs using artificial intelligence

#### 2.5.1. Existing deep learning-based segmentation tools for fluorescence microscopy.

**Fig 5:**
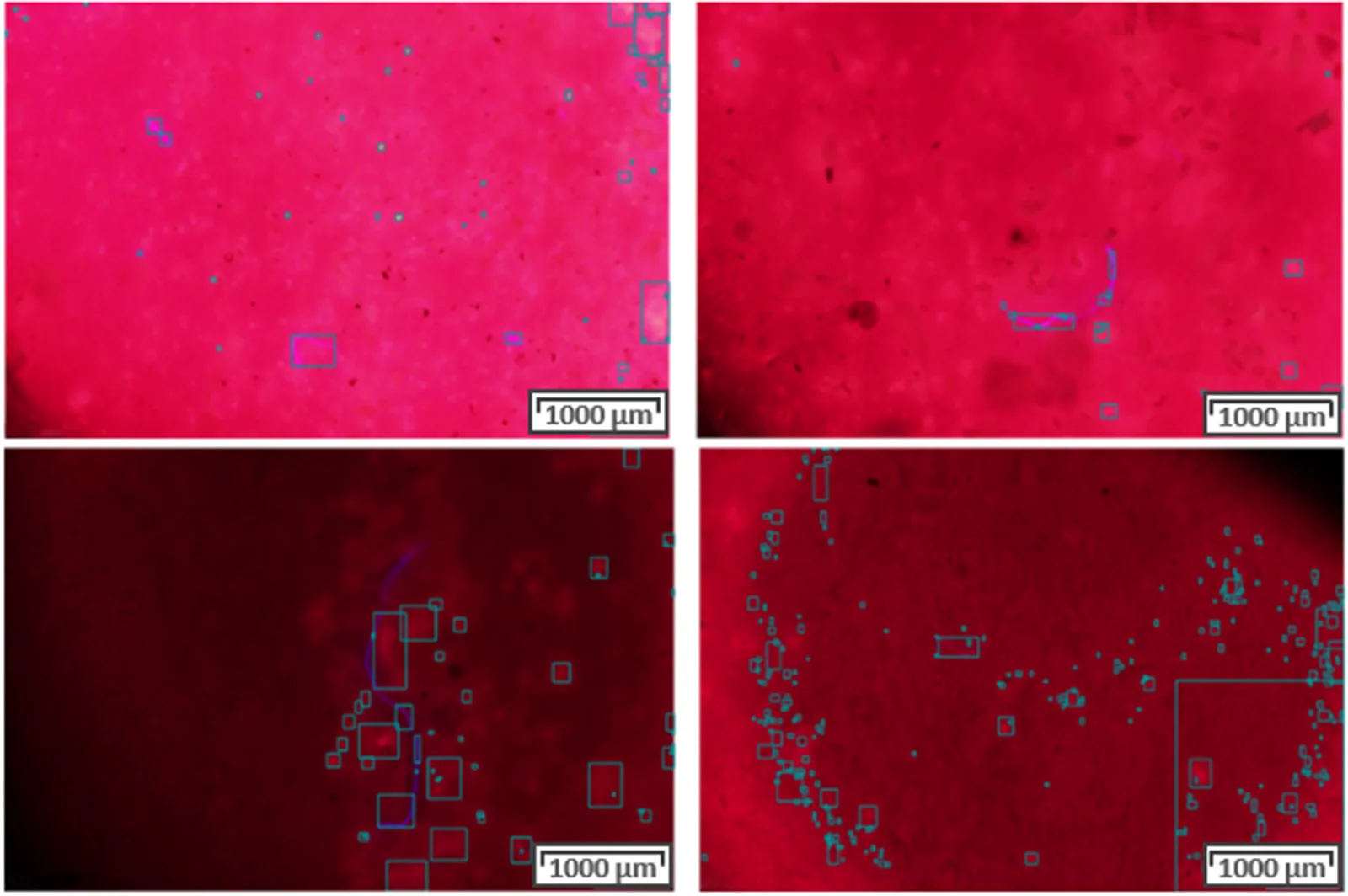
Example outputs from the MP-VAT2 tool across four representative image profiles, showing different illumination and background conditions (4x objective). Detected particles are outlined by small colored squares, illustrating over-detection under uneven illumination.

Preliminary tests were run using state of the art (SOTA) methods using the Microplastics Annotation Package (MAP) tool from MP-NET [20] and MP-VAT2 (Microplastics Visual Analysis Tool 2) [15] on our in-house dataset. MPs were substantially over-detected when using automatic image thresholding methods (MP-VAT2), notably due to a non-uniform illumination leading to the detection of many noise particles (Fig 5).

**Fig 6:**
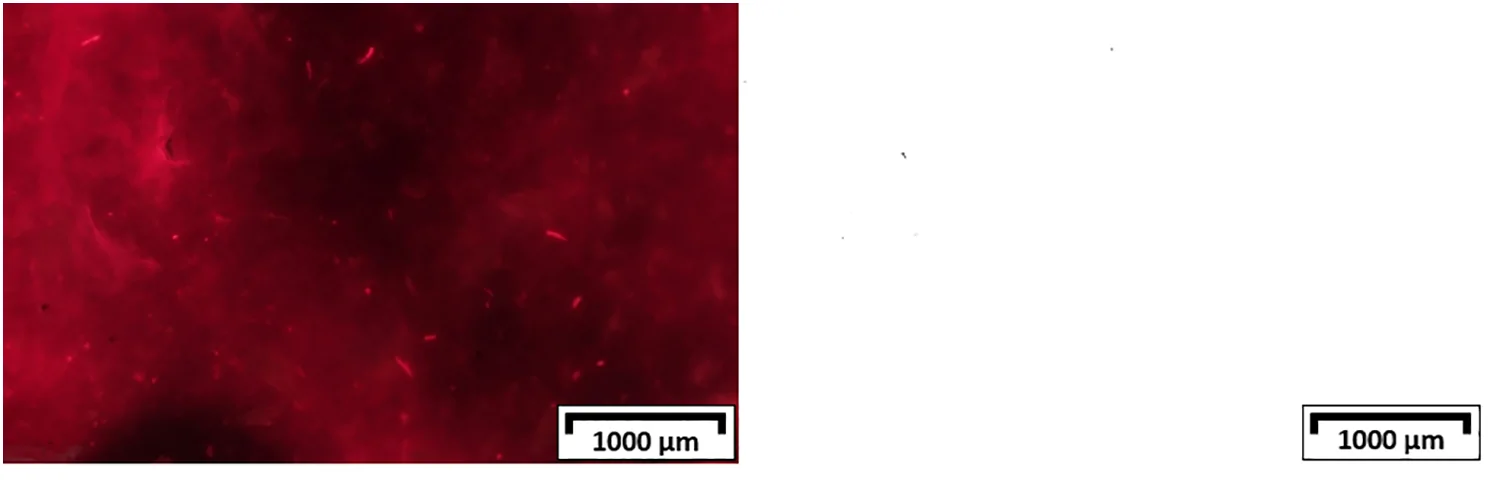
Example of MP-NET predictions on an in-house image (x4 objective). On the left, the input fluorescence image shows bright MP blobs. On the right, the mask prediction generated by the MP-Net tool. The nearly plain white mask indicates that most particles were classified as background (black = foreground), revealing a data drift issue between the original MP-SET training images and our dataset (different color and brightness profiles).

When using the pre-trained deep learning model MP-NET, the mask prediction was almost plain white meaning that particles were considered as background (foreground was black, Fig 6). In this case, many MPs are missed due to data drift. Instead of having bright yellow MPs as in MP-SET, they appeared white or blue in the in-house dataset. Fine-tuning the model on our dataset was necessary to cope with this data drift (see section 2.5.3).

#### 2.5.2. Semi-automated annotation clustering and Labkit.

Interactive segmentation was performed with Labkit (version 0.1.17) [36], a Fiji plugin for manual and automated image annotation. A lightweight pixel classification model was trained on the fly based on user-provided scribbles. This model classifies each pixel as foreground or background using simple features such as image gradients, which capture local changes in intensity, and HOG (Histogram of Oriented Gradients) descriptors, which encode local edge orientations [37]. This allows fast training, making it well suited for online training, but it also limits its generalization across images, so that two images in the same batch may not be properly annotated without overfitting with many scribbles. To mitigate these limitations, batches of 20 images were grouped based on similarity, as described in the next section. In addition, GPU acceleration was used when available (requires Nvidia GPU), and image resolution was reduced by a factor of 2 without loss of mask quality. Hardware constraints and Labkit memory management also dictated the maximum number of images that could be loaded at once based on their resolution. The annotation procedure using Labkit software (installed as part of the Fiji plugin ecosystem) is detailed in S1 Protocol (see Fig A therein for an example of the annotation).

Images were grouped by similarity using recent Contrastive Language-Image Pre-training (CLIP) [38] image embeddings. We used Uniform Manifold Approximation and Projection (UMAP) [39] to visualize image clusters. The Fiftyone framework [40] was used to conduct cluster image analysis. Four sets of images were captured at different times over the year. Images were clustered in 15 groups for each set and batches of maximum 20 images per group were created. This sampling protocol produced images similar enough for Labkit batch annotation while covering diverse images across clusters. Although this procedure significantly accelerates the measurements of MPs contamination, it is a time-consuming manual work to annotate the full dataset (6711 images in total). We only sampled the data once per set, which enabled us to annotate nearly 10% of the entire dataset. In this article, we show that is enough to train a U-Net model with human-level performance for detecting MPs on this type of data. The complete data preparation pipeline is shown in Supplementary S2 Fig. This framework allowed efficient processing of the large dataset (13,402 raw images) while maintaining high-quality annotations for training the U-Net model.

#### 2.5.3. Fully-automated annotation using U-Net.

Model selection. Inspired by the work of Park et al. [20], we adopted a similar U-Net-based [41] model for our experiment. We chose another implementation based on a MMsegmentation [42] framework as it allows us to experiment with SOTA model architecture such as Transformers [43] later on. The U-Net architecture comprised two main parts (S3 Fig):

- Contracting Path: This initial part of the network acts as an encoder, capturing contextual information from the input image. It consists of convolutional and pooling layers, reducing the spatial dimensions while increasing the depth of feature maps.
- Expanding Path: Following the contracting path, this part served as a decoder. It involves upsampling and concatenation operations to recover spatial information lost during the contracting phase. It reconstructs a segmentation map with pixel-level accuracy.

The architecture features skip connections between corresponding layers in the contracting and expanding paths. These connections facilitate the flow of detailed information across the network, aiding in precise segmentation. It won the Cell Tracking Challenge at ISBI 2015 in the two most challenging transmitted light microscopy categories (Phase contrast and DIC microscopy) by a wide margin and is still widely used for biomedical segmentation thanks to its good performance and low complexity. Compared to more recent architectures such as transformers, U-Net is lightweight and can be trained on average consumer laptops with 8 Go of VRAM.

Model fine-tuning. Park et al. [20] showed that their U-Net variant trained using Dice loss [44] and Stochastic Gradient Descent (SGD) [45] was the most successful in terms of mIoU and mF1-score. Hence, we likewise kept those two parameters. The main difference compared to our implementation is the default backbone as used in Ronneberger et al. [41] compared to the Resnet101 backbone used by Park et al. [20]. We also use 400 x 400 input size instead of 256 x 256 image patches to have large enough windows without degrading the image resolution too much. Traditional random resize, random image flip and photometric distortion are applied as data augmentation. For reproducibility, the configurations for data augmentation and optimizer settings are provided respectively in S2 and S3 Tables. It is worth noting that our model uses a lightweight backbone compared to Park et al. [20] which allows us to have larger patch size.

Model inference. Segmentation masks produced by our model are transformed into instance segmentation using Connected Components Labeling (CCL) from OpenCV. A score based on the relative contrast of the MP has been established to filter out particles with low fluorescence. This score was calculated based on image contrast (Equation 1), and could be adjusted on different data partitions (*i.e.,* Viscera and Gills) to remove low-contrast particles.


$$\begin{array}{c} C_{RGB}=\;\sqrt{{\sum_{c\in \{R,G,B\}}\left(\frac{\text{1}}{\left|\Omega_{f}\right|}\sum_{p\in \Omega_{f}}I_{c}(p)-\frac{\text{1}}{\left|\Omega_{b}\right|}\sum_{p\in \Omega_{b}}I_{c}(p)\right)}^{\text{2}}} \end{array}$$ (1)

Where $\Omega_{b}$ and $\Omega_{f}\;$represent the background and foreground, respectively; I_*c*_(p) is the intensity of pixel p in color channel c∈{R,G,B}; and the sums are taken over all pixels in the corresponding regions.

#### 2.5.4. Evaluation metrics.

In assessing the performance of our segmentation models, a comprehensive set of three metrics was employed: precision (Equation 2), recall (Equation 3) and F1-score (Equation 4). Precision quantifies the model’s ability to correctly identify positive instances among all instances classified as positive. Recall, also known as sensitivity, gauges the model’s capability to correctly identify all positive instances within the dataset. F1-score serves as a harmonic mean of precision and recall, offering a balanced assessment of a model’s performance. All three metrics range from 0 to 1, with values closer to 1 indicating better model performance.


$$\begin{array}{c} \text{Precision }=\frac{TP}{TP+FP^{\prime }} \end{array}$$ (2)

$$\begin{array}{c} \text{Recall }=\frac{TP}{TP+FN^{\prime }} \end{array}$$ (3)

$$\begin{array}{c} \text{F1}-\text{Score}=\;\frac{\text{2}\;*\;Precision\;*\;Recall}{Precision\;+\;Recall} \end{array}$$ (4)

Where TP represents true positives, TN represents true negatives, FP represents false positives, and FN represents false negatives. This metric offers insight into the model’s pixel-level performance, crucial for evaluating segmentation tasks accurately.

In Park et al. [20], the authors showed no performance improvements when using TTA (test time augmentation), so we conducted our evaluations without TTA as well. Annotated data is split such that 70% is used for training and 30% for testing.

#### 2.5.5. Evaluation protocols.

Dataset distribution. The entire dataset was annotated using both semi-automatic and fully automatic models. U-Net model is used to count MPs in unlabelled data while semi-automatic models with manual corrections are used to annotate training and tests subsets using the method described in section 2.5.2. Table 3 reports the amount of data used for the different islands and different sample matrices for each subset.

**Table 3:** Image dataset distribution across islands and sample type (BENI and CBENI), with assignment to training, test, and unlabeled subsets.

Partitions	Total	Train	Test	Unlabeled
Makemo	2510	152	66	2292
Hao	1814	37	17	1760
Tubuai	2387	194	85	2108
BENI total	5985	371	162	5452
CBENI total	726	12	6	708
Overall Total	6711	483	168	6160

U-Net model evaluation. A single U-Net model was trained and evaluated for all islands and both sample types (BENI and CBENI) using the segmentation metrics described in section 2.5.4. CBENI had a small amount of training and testing data due to our sampling strategy but this did not affect the model’s ability to segment any sample matrix.

Human level evaluation. Model performance was compared to that of a human annotator. A set of 100 random test images was selected, and another expert annotated them using Labkit. This allowed a direct comparison of U-Net and human performance on the same images.

µFTIR evaluation. In order to identify plastic polymer present in our samples and to cross-validate the NR-based detection, additional giant clam samples were analyzed by µFTIR (n = 29 corresponding to 13 Gills, 13 Viscera and 3 blanks). The sample particles were characterized by an imaging technique based on a focal point array (FPA), using the Spotlight 400 FTIR-IR microscope (Perkin Elmer), at the Laboratoire de Chimie de l’Environnement (UMR LCE-7376). In reflectance mode, the entire surface of the stainless-steel filters was scanned from 4000 to 650 cm^-1^ with a spectral resolution of 16 cm^-1^ and a pixel size of 25 µm [46]. The siMPle (Systematic Identification of MicroPlastics in the Environment [47]) software was then used to characterize the particles (i.e., size, volume, mass) and identify the polymer type, by comparing the sample spectrum to spectra in the MPHunter reference spectra library [48]. These comparisons were performed over the regions from 3600 to 2600 cm^-1^ and from 2280 to 704 cm^-1^, with a minimum match score of 60%. A minimum particle-size threshold of three contiguous pixels was applied during image segmentation in siMPle to reduce false detections. In order to characterize the shape of the analyzed MPs, a categorization into fiber and fragment was performed according to the ratio between particle length and thickness, with a classification as fiber for a value greater than 3, and as fragment for lower values [49].

### 2.6. Data analysis

Statistical analyses were performed using R Studio software (2025.09.2). Count data from the automatic model and µFTIR analyses were corrected for potential contamination in controls and normalized to organ weight, so that MP concentrations are expressed as the number of particles per gram of wet weight (particles·g^−1^ ww).

To assess differences in total MPs accumulation between gills and viscera of the giant clam across islands, permutational univariate analyses of variance (aovp, lmPerm) were performed separately for each detection method, including Island, Station, and their interaction (Island × Station) as fixed factors (α = 0.05, 1000 permutations). To assess the digestion effect on polymer integrity (length and width), the same permutational ANOVA approach was applied, with digestion step as a fixed factor and polymer type treated separately (n = 3 per polymer and step). Significant effects were further explored with post-hoc pairwise comparisons between islands.

Additionally, the relationship between MP abundance, size and shape obtained by the automatic model and µFTIR was assessed using Spearman’s rank correlation coefficient, providing insight into the monotonic association between the two methods, independent of absolute counts. This approach allowed us to evaluate both method-specific differences and overall trends in particle detection.

## 3. Results

### 3.1. Optimizing digestion protocols for MPs extraction from giant clams

With the aim of assessing the efficiency of digestion protocols for giant clam *viscera*, several experimental conditions were tested, including variations in time, assistance methods, and sequential chemical treatments. Standard KOH digestion (10%, 40°C, 72 h) left substantial organic residues after filtration, indicating incomplete tissue removal (photos A1–B1; Table 4). Extending the digestion time following Zhou et al. [11] slightly reduced the amount of organic material, but filters remained overloaded and the protocol was time-consuming, with 24 h at 40°C followed by 7 days at room temperature (photos A2–B2; Table 4). Independently, the effect of physical assistance for KOH digestion was evaluated to determine whether additional mechanical energy could enhance tissue digestion. Ultrasound (30 min) combined with a brief microwave treatment (500 W, 3 min) [12,31] further decreased organic residues compared to standard KOH digestion, resulting in visibly cleaner filters; however, complete digestion was not achieved (photos A3–B3; Table 4). Finally, double digestion approaches produced contrasting outcomes depending on the chemical used. KOH followed by H_2_O_2_ treatment [4] led to incomplete tissue removal and partially discolored remaining material, suggesting only a modest improvement in digestion efficiency (photos A4–B4; Table 4). In contrast, a second digestion step using 20% HNO_3_ [32] after KOH treatment effectively removed visible organic matter (photos A5–B5; Table 4), producing cleared filters suitable for downstream analysis. This protocol was therefore selected for subsequent analyses.

**Table 4:** Visual comparison of filters obtained from different digestion protocols tested on giant clam viscera, under macroscopic (x0) and microscopic (x40) magnification (Olympus IX73). The initial 10% KOH digestion [30,50] (A1–B1) left substantial organic residues in viscera. Extending digestion time [11] (A2–B2) slightly improved tissue degradation. Ultrasound and microwave assistance [12,31] (A3–B3) reduced the organic load but did not ensure full digestion. Double KOH–H_2_O_2_ digestion [4] (A4–B4) partially discolored remaining tissue, while the second digestion with 20% HNO_3_ [32] (A5–B5) effectively removed residual organic matter, yielding clear filters suitable for analysis.

Digestion method	Simple digestion KOH 10%, 40°C, 72h	Simple digestion KOH 10%, 40°C, 24h + 7 days at room temperature	Ultrasound (30 min) + KOH digestion (10%, 40°C, 72 h) + Microwave (500 W, 3 min)	Double digestion (KOH 10%, 40°C, 72h + H_2_O_2_ 30%, 40°C, 2h)	Simple digestion KOH 10%, 40°C, 48h + HNO_3_ (1h, room temperature)
**Reference**	[30,50]	[11]	Adapted from [12,31]	[4]	Adapted from [32]
**Macroscopic view**	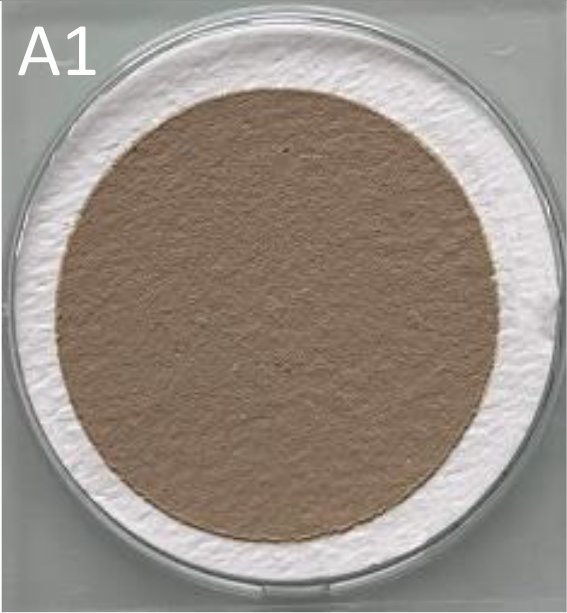	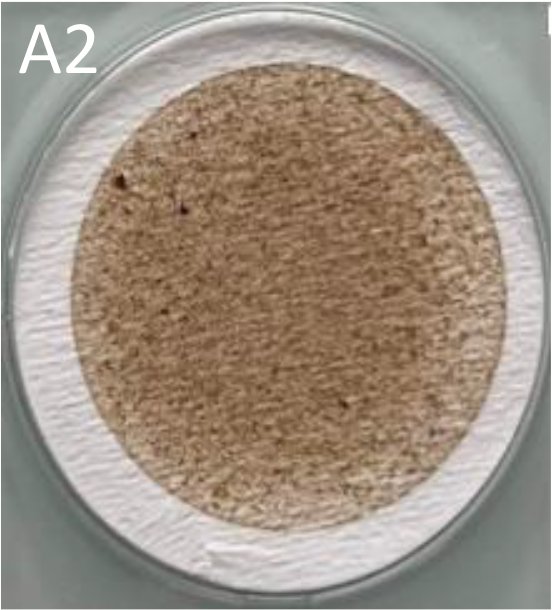	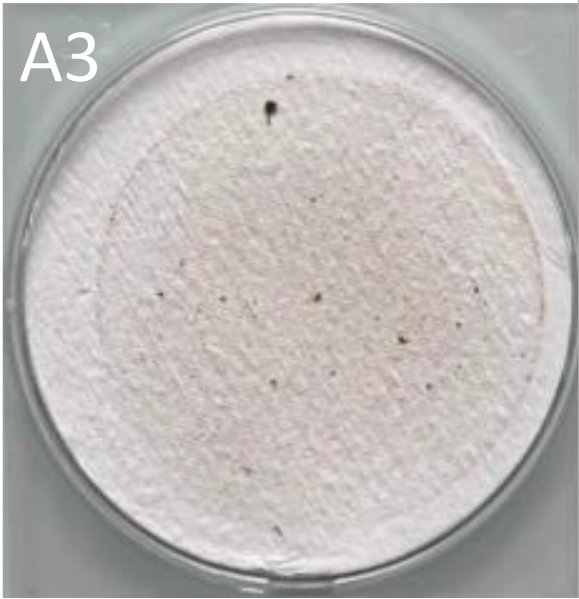	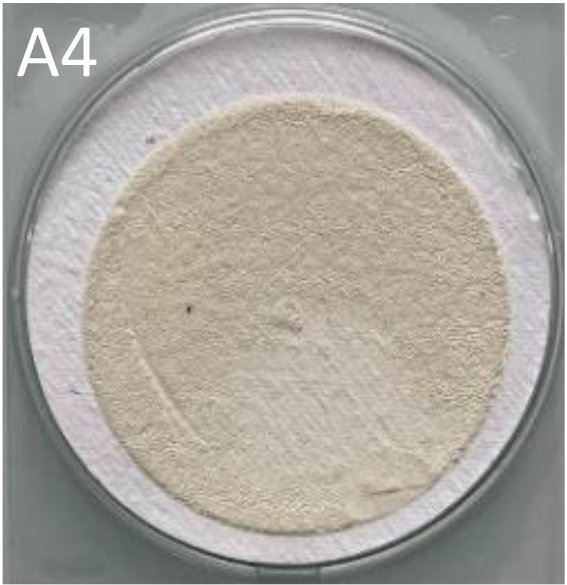	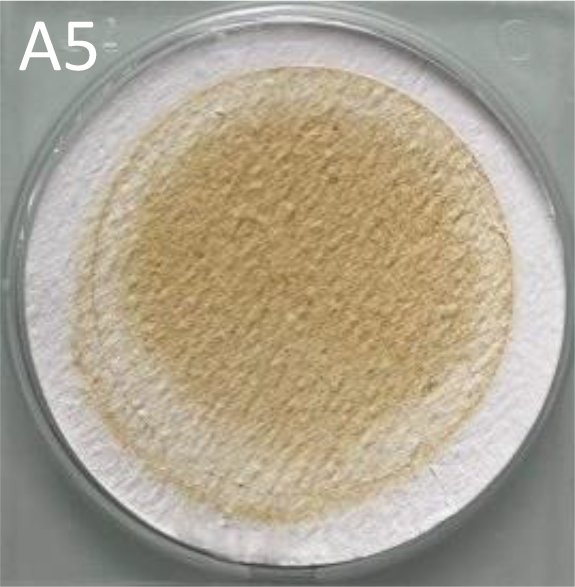
**Microscopic view**	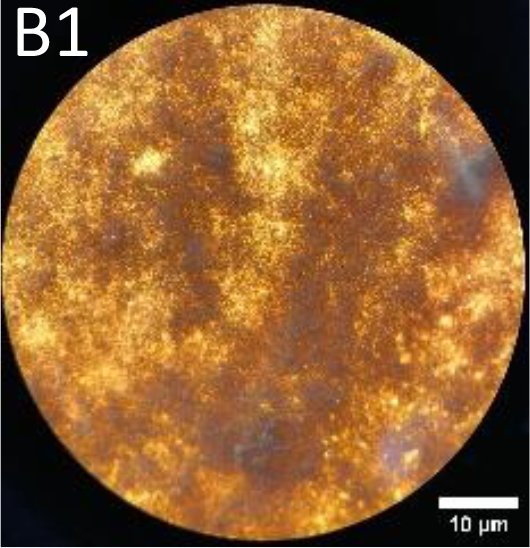	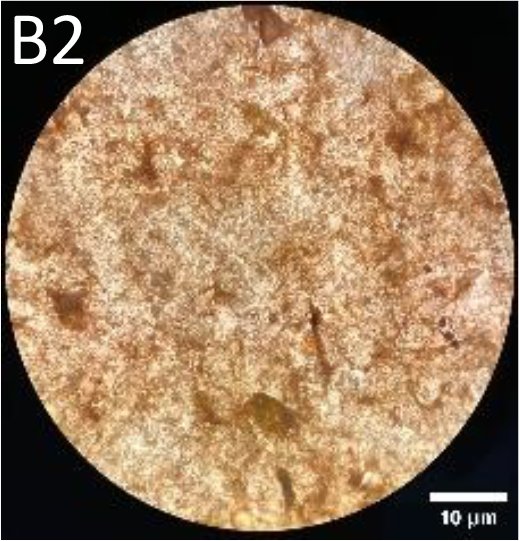	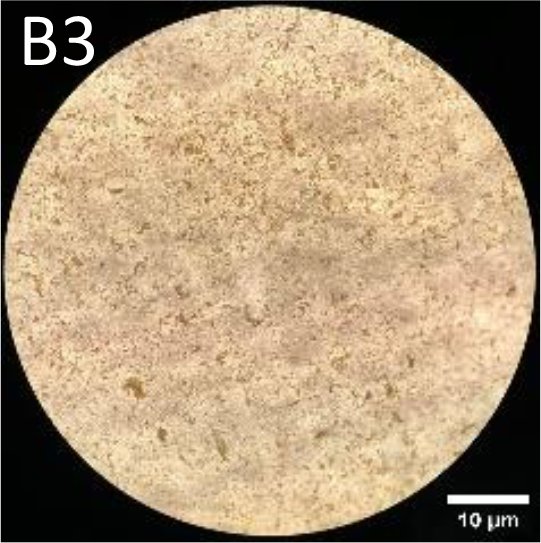	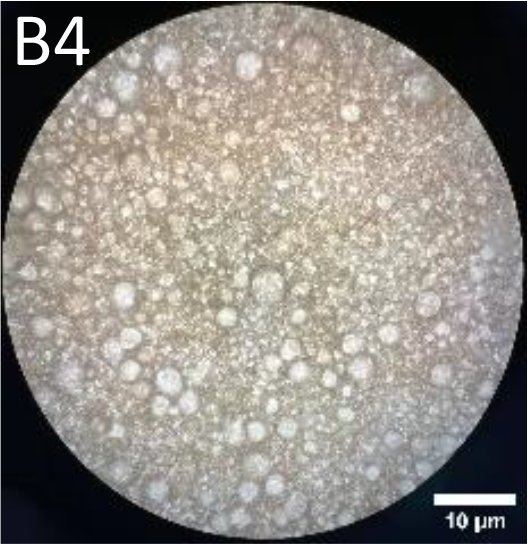	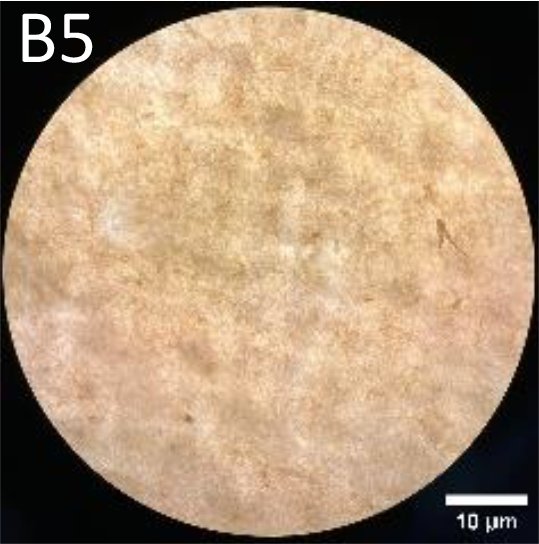

Recovery tests performed on five polymers (PP, PE, PVC, PA, and the PE/PP co-polymer) indicated that all polymers were consistently recovered across digestion steps. No visible alterations were observed for most polymers (S4 Fig), although slight opacification and curling were noted for PA. Quantitative analyses showed no statistically significant differences in particle width or length following digestion (S5 and S6 Figs; aovp, p > 0.05).FTIR spectra showed no major changes for PP and PVC, while PE exhibited a minor peak at 1355 cm^−1^, consistent with secondary alcohol formation, and a weaker band at 1748 cm^−1^, indicative of carbonyl groups and mild oxidation. PA spectra displayed an increased baseline and higher background absorbance between 500 and 1500 cm^−1^, with a decrease in overall transmittance, although the main spectral features remained comparable to the control (S7 Fig).

### 3.2. U-Net Model evaluation

The machine-learning performance metrics were high, as reported in Table 5. All model evaluation metrics were above 0.513. The F1-scores, which combine precision and recall to assess the model’s accuracy at pixel-level segmentation, were 0.741 for the BENI samples and 0.657 for the associated controls (CBENI). For comparison, a human annotator achieved an F1-score of 0.68. These values indicate that our model performs at or slightly above human-level accuracy for detecting fluorescent particles, suggesting reliable segmentation performance. For a direct visual comparison of the original fluorescence images, human annotations, and U-Net predictions for the same ROI, see S8 Fig.

**Table 5:** Precision, recall and F1-score results for each sample type. Human annotator tested on a 100-image subset of the BENI test set.

Samples	n (test images)	Precision	Recall	F1-score
BENI	162	0.658	0.848	0.741
CBENI	6	0.513	0.912	0.657
Human (BENI only)	100	–	–	0.680

**Fig 7:**
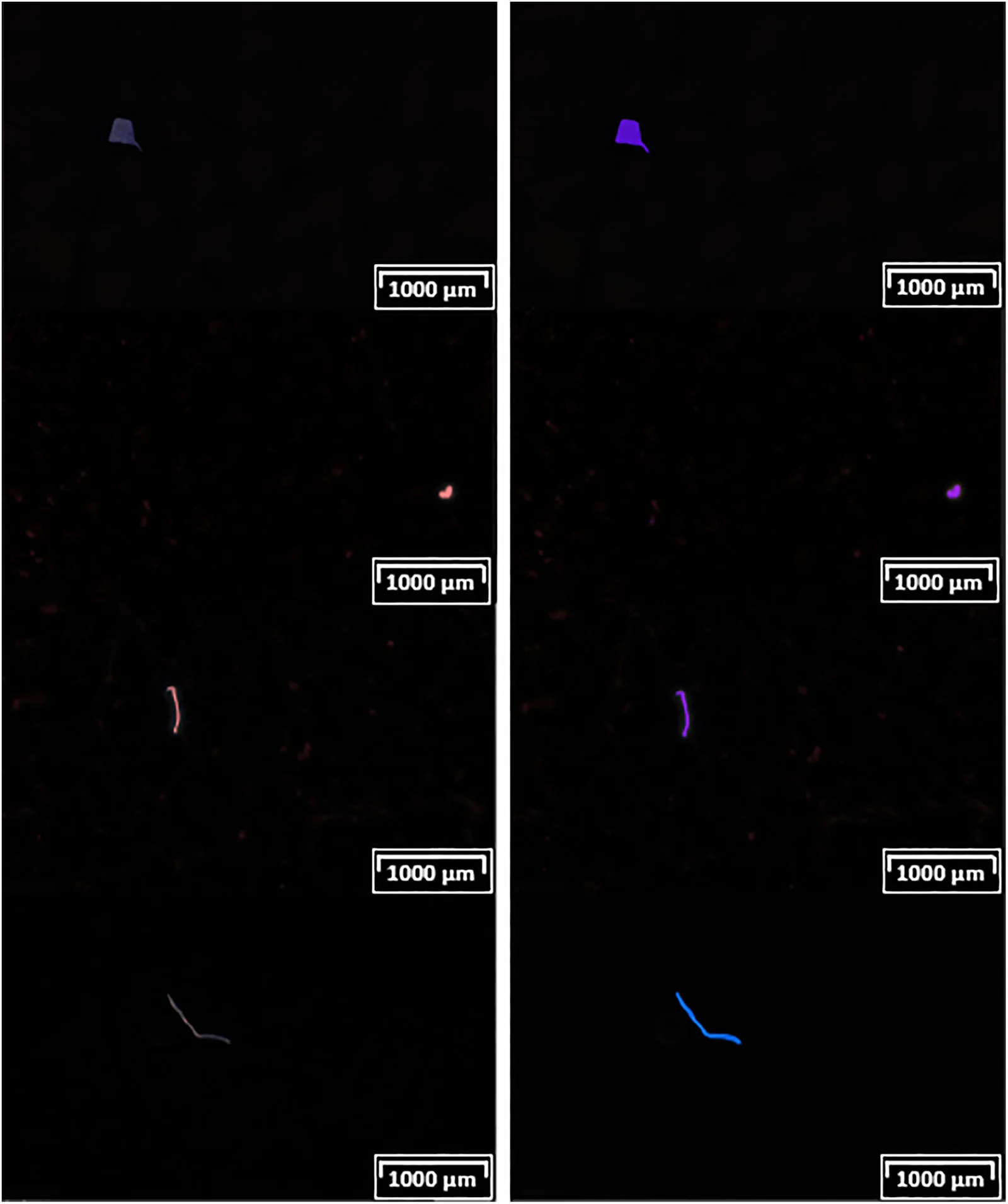
Example of good detections by the automatic counting model, on particles of varying nature. Photos on the left correspond to the composite image, and those on the right to the model detection. Particles were extracted from giant clam viscera and gills collected in Tubuai and Hao. Photographs were taken under an epifluorescence microscope (Olympus IX73) equipped with an Olympus® DP74 Color camera. Observations were made at x4 magnification.

An additional qualitative analysis of the proposed model was also performed to better identify the limits of the model. The following results illustrate the particles detection capability of the model without the application of a score (as a reminder, based on the relative contrast of the particle). Here, the automatic detection model enables particles of various characteristics to be detected effectively: fluorescence color and intensity (blue or red), size (from 20 µm to >1000 µm) and particle shape (fragment or fiber) (Fig 7).

**Fig 8:**
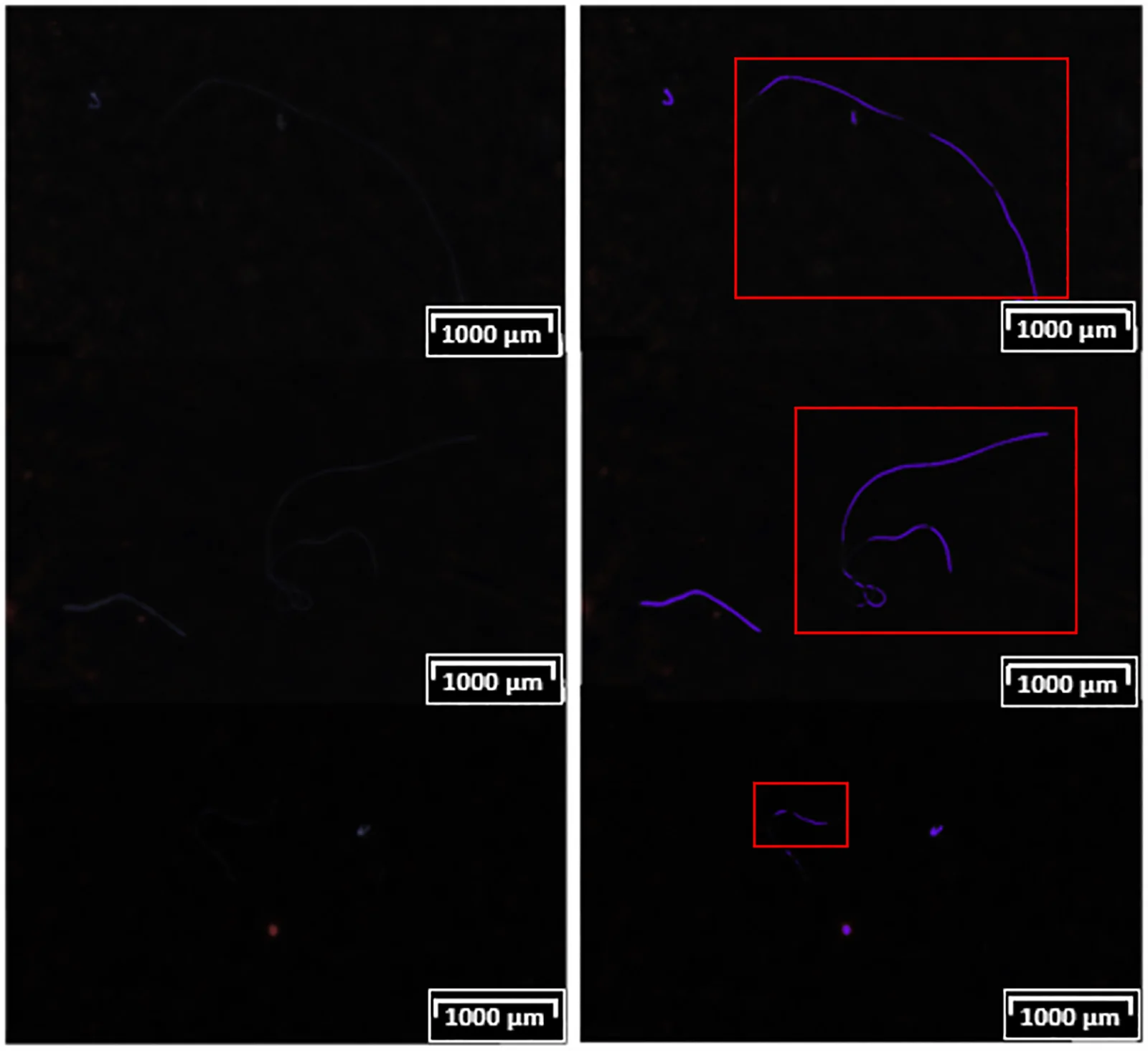
Example of false detections by the automatic counting model, on particles extracted from the gills and viscera of giant clams (collected in the islands of Hao and Makemo). Photos on the left correspond to the composite image, and those on the right to the model detection. Red boxes highlight examples where a single fiber was incorrectly segmented into multiple detections due to heterogeneous fluorescence within the particle. The photographs were taken under an epifluorescence microscope (Olympus IX73) equipped with an Olympus® DP74 Color camera, at x4 magnification.

On the other hand, in rare cases, the model showed some limitations. Particles with low fluorescence intensities could lead to over-detection. Sometimes, even the human eye had trouble distinguishing the particle fluorescent response whereas the model was able to detect it well, as shown in Fig 8. In addition, for some particles whose fluorescence levels were not homogeneous at the particle scale, some parts may not be detected, leading to the detection of a single particle as multiple-parts and an overestimate of the number of particles detected (Fig 8).

### 3.3. Contamination evaluation in giant clam

**Fig 9:**
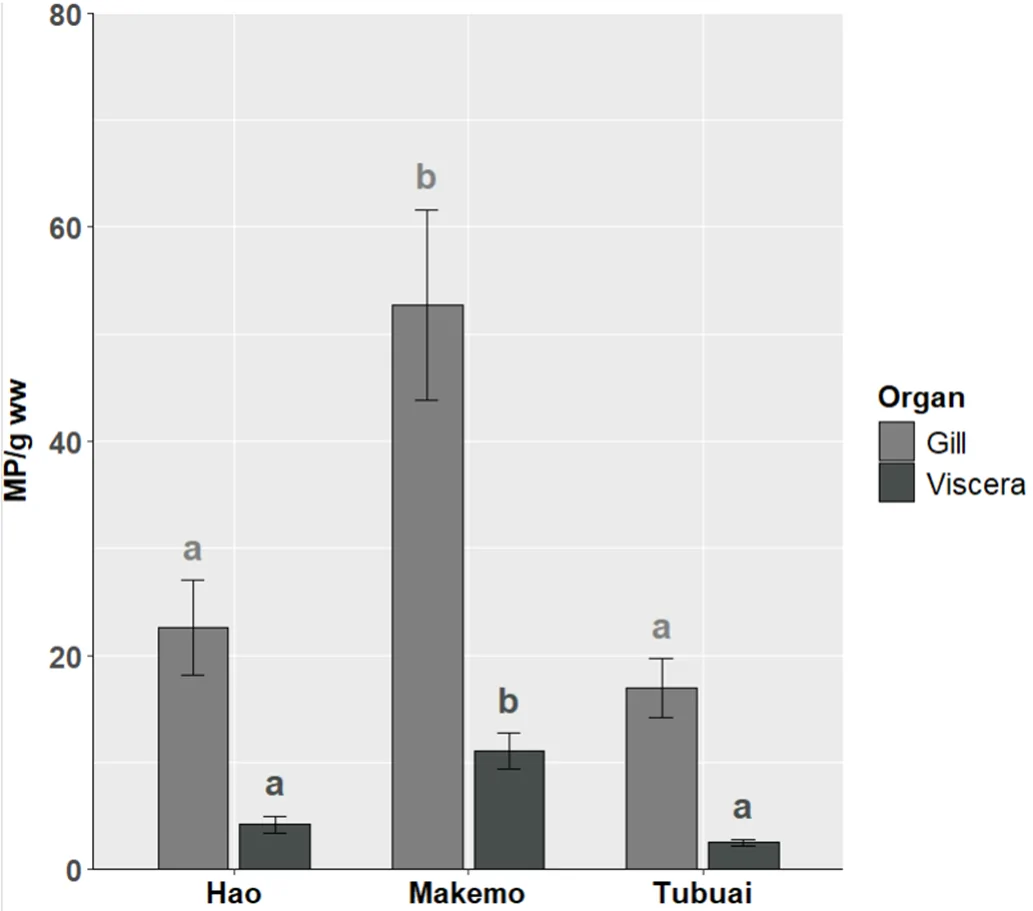
Particles concentration measured in the giant clams (gills and viscera) from Hao, Makemo and Tubuai and detected by the NR method (mean ± se) (n = 15 per island). Different letters above bars indicate significant differences between islands for each organ (Gill or Viscera), as determined by pairwise permutation t-tests with false discovery rate (FDR) correction). Data shown have not been corrected using the score-based threshold (cf Equation 1).

Levels of contamination quantified within the giant clam were obtained from data segmented automatically by our model (“unlabeled”), except for data segmented manually (which form part of the training and test subsets). These results revealed differential levels of contamination in giant clams depending on the organs considered (aovp, p < 0.0001), but also on the islands studied (aovp, p < 0.0001). The highest contamination was recorded in gills, ranging from 16.90 ± 2.72 particles.g^-1^ ww in Tubuai to 52.72 ± 8.86 particles.g^-1^ ww in Makemo (Fig 9). A similar but lower pattern of contamination was observed in viscera, with concentrations varying from 2.50 ± 0.29 particles.g^-1^ ww in Tubuai to 11.02 ± 1.67 particles.g^-1^ ww in Makemo (Fig 9). At the pooled-organ scale (i.e., gills and viscera), contamination ranged from 3.87 ± 0.30 particles g^−1^ ww in Tubuai to 17.5 ± 2.15 particles g^−1^ ww in Makemo.

**Fig 10:**
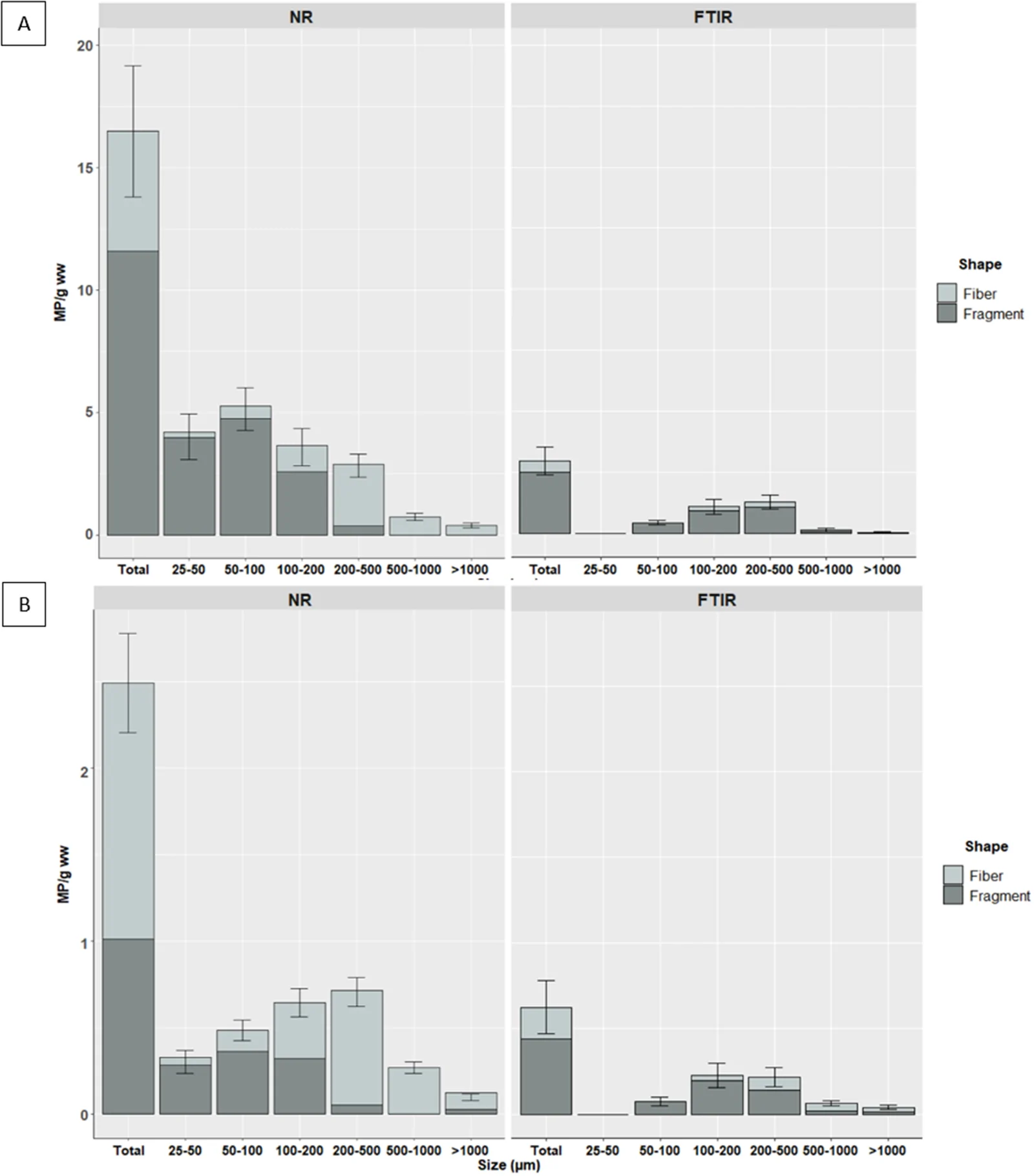
Particles accumulation profiles by shape and size in *T. maxima* gill (A) and viscera (B) collected in Tubuai, and detected by NR and µFTIR analysis methods (n = 15 by organs for NR, and n = 13 for µFTIR). Bar charts represent mean values ± standard error (SE). Note that Y-axis scales differ between gills and viscera.

To cross-validate the NR-based ML approach, additional samples from Tubuai were analyzed using µFTIR for particles larger than 25 µm. Overall, MPs levels quantified in the different organs by µFTIR were significantly lower than those measured with NR staining (aovp, p-value < 0.001), but gills showed the highest level of contamination for both methods (Fig 10). The concentrations obtained by µFTIR were equal to 2.95 ± 0.56 particles.g^-1^ ww for gills and 0.62 ± 0.15 particles.g^-1^ ww for viscera (corresponding to 0.80 ± 0.16 particles.g^-1^ ww at pool scale). NR overestimation was more pronounced in gills than in viscera (Fig 10). In gills, fragments predominated (11.58 ± 1.79 particles.g^−1^ ww NR vs 2.49 ± 0.54 particles.g^−1^ ww with µFTIR) over fibers (4.90 ± 0.95 particles.g^−1^ ww with NR vs 0.46 ± 0.11 particles.g^−1^ ww with µFTIR, Fig 10A), with again similar trends for both methods (Fig 10A). In viscera, µFTIR showed more fragments than fibers (0.44 ± 0.12 particles.g^−1^ ww vs 0.18 ± 0.04 particles.g^−1^ ww), whereas NR detected more fibers (1.48 ± 0.14 particles.g^−1^ ww) than fragments (1.01 ± 0.16 particles.g^−1^ ww, Fig 10B). Regarding particle size, the 25–50 µm and 50–100 µm classes were more abundant with NR than µFTIR (Fig 10). Overall, Spearman rank correlation analyses revealed no significant relationship between NR particle counts and µFTIR-confirmed polymer abundance by organ (gills or viscera) (S4 Table). Similarly, no significant correlations were observed when analyses were stratified by particle morphology (fibers vs fragments) or by particle size classes (S4 Table).

**Fig 11:**
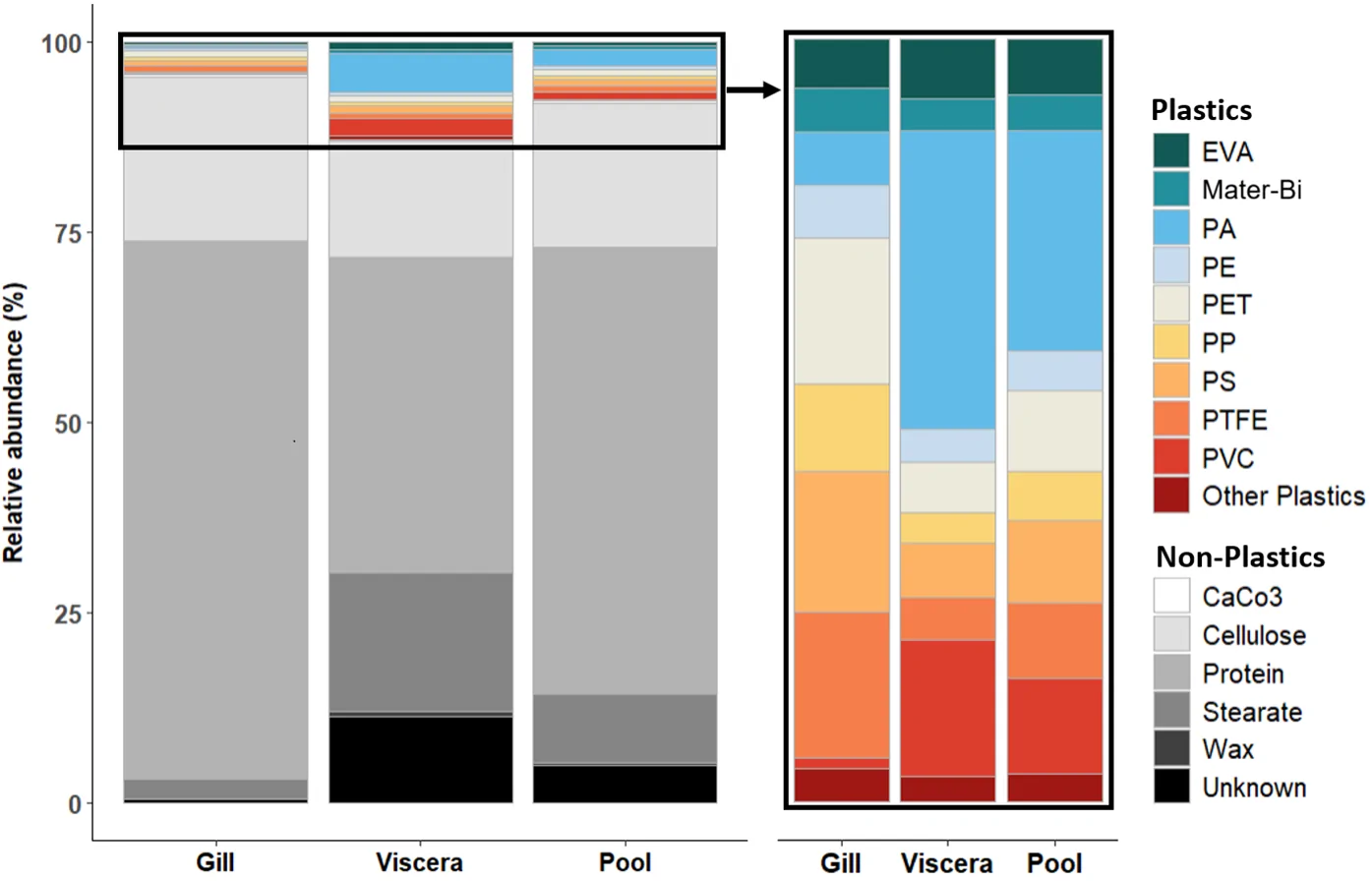
Relative abundance of polymers and natural materials in *T. maxima* pool (gills and viscera) identified by µFTIR (n = 13 by organs). Quantified plastics, synthetic and natural materials (“non-plastics”), as well as unidentified particles are listed. The “Other Plastics” category includes polymers with an abundance of less than 2% (i.e., Rosin, Silicone, Polyurethane, and Polyisoprene). EVA: ethylene-vinyl acetate; PA: polyamide; PE: polyethylene; PET: polyethylene terephthalate; PP: polypropylene; PS: polystyrene; PTFE: polytetrafluoroethylene; PVC: polyvinyl chloride; CaCO_3_: calcium carbonate.

The µFTIR analysis highlighted the abundance in our samples of proteins, cellulose and stearates (Fig 11). Concerning their proportion, gills contained more proteins (70.7% in the gills, 41.6% in the viscera) and cellulose (21.5% in the gills, 15.3% in the viscera). However, the viscera contained a much higher proportion of stearates than the gills (18.1% vs. 2.7%). Generally, fibers were over-detected with NR staining compared to µFTIR, especially in the viscera. Some size classes were overestimated too by NR, such as the 25–50 µm size class (reaching nearly 24.3% in the gills and 12.1% in the viscera in NR; and absent in µFTIR), and the 50–100 µm size class (31.3% in the gills and 19.4% in the viscera in NR vs. 14.6% and 11.3% in µFTIR). Regarding the types of plastics found especially in the pool (gill and viscera), PA (28.9%) were predominantly found, followed by PVC (12.6%), PS (10.7%), PET (10.6%) and PTFE (9.9%). Unidentified compounds accounted for 0.4% of particles analyzed in gills and 11.3% in viscera.

## 4. Discussion

This work led to the development of an original quantitative approach based on the NR detection method, whose characteristics (low cost, ease of implementation) make it very popular for use in isolated island systems and, more generally, in low – and middle – income countries. The automatic counting model we developed was inspired by the methodology of Park et al. [20], but it offers additional advantages, particularly in handling large volume of data. This required the implementation of an active learning strategy, followed by a semi-automatic model for interactive annotation using Labkit. NR staining allowed the detection of notable levels of particles across islands and within individual organs. To gain a more complete understanding of methodological performance and potential biases, µFTIR spectroscopy was applied as a complementary approach. Comparison between the two methods indicated that some particles detected by NR were not confirmed as plastics by µFTIR, and that contamination patterns can differ depending on the method. These observations highlight the importance of careful methodological consideration, which is explored in detail in the following sections, covering sample composition, model calibration, optical setup, NR advantages, µFTIR limitations, and environmental contamination patterns.

### 4.1. A new quantitative approach using NR detection method

#### 4.1.1. Model overview and dataset characteristics.

The model developed in this study enabled the effective and automatic quantification of fluorescent particles with diverse profiles within our samples. A key advantage of this model lies in its compatibility with an acquisition protocol that incorporates multiple fluorescence filters. This approach allows for the detection of a broader range of plastic polymers, thereby complementing the polymer types typically detected. Indeed, compounds such as PVC, PA, PLA and even HDPE [18] are compounds not detected using the commonly employed FITC fluorescence filter [17]. However, these polymers can be detected through the use of TRITC (orange) and DAPI (blue) filters [16,17]. Furthermore, this model was based on an exceptionally large dataset (6711 photos) with diverse image profiles. By contrast the MP-Set dataset [20] – the only previously published dataset for MP characterization in fluorescence microscopy images, to our knowledge – comprises only 99 high-resolution images (1280 x 960–7140 x 5424 pixels), where MPs appear as very small spots covering just 0.015% of the total pixel number. Mask annotations were obtained by manual thresholding methods using ImageJ, and refined with pixel-wise majority voting from three independent annotators to improve accuracy. In contrast, our dataset and approach offer greater scale and heterogeneity, as well as reduced subjectivity, through the use of active learning and semi-auto models for interactive annotation using the Labkit tool.

A recent multispectral approach, FIMAP [51], reports high performance for NR–based MP detection and identification (precision 90%, recall 100%, F1 94.7%, mean IoU 87.7%). It is important to consider the experimental conditions underlying these metrics: they were obtained on a controlled benchmark consisting of ten pristine virgin polymers larger than 3.14 mm, manually arranged without overlap on clean filter paper, and calculated at the object level for the combined task of detection and polymer identification rather than as a pixel-level segmentation score. In this controlled set, performance decreased for smaller particles; for the 35–104 µm fraction, the authors reported reduced classification accuracy, with several polymer pairs becoming statistically indistinguishable. When the method was subsequently applied to a realistic matrix (Fenton-treated biosolids), the workflow was adapted accordingly, including re-tuning of the segmentation pipeline and reporting of suspected particles (>24.6 µm), while the authors indicated that polymer identity required further validation, particularly for smaller particles. Ho et al. [51] therefore provide an important methodological benchmark under controlled conditions rather than a directly comparable environmental benchmark. Our study addresses a more complex environmental context, with weathered and overlapping MPs ranging from 20 µm to more than 1000 µm, embedded in real giant-clam tissue with organic-matter interference, evaluated at the pixel level (F1-score = 0.741) and cross-validated against µFTIR polymer identification. At the same time, the two studies converge methodologically: FIMAP’s 20-channel multispectral acquisition, developed to overcome single-filter thresholding limitations, mirrors the rationale for our multi-spectral (DAPI and TRI) composite, and its explicit minimum particle-size filtering parallels the small-detection removal step in our pipeline.

These interactive pixel classification methods, commonly used in digital pathology for interactively segmenting cells or tissues from microscopy images, were applied here for the first time in MP research. These versatile tools benefit from the broader research effort in biological image analysis, and enhance both annotation consistency and model generalization. Regarding performance, our model achieved F1-scores of 0.741 for BENI and 0.657 for CBENI, with BENI showing a slightly higher F1-score than the MP-Net tool (F1-score = 0.736 [20]). Furthermore, when compared with manual human annotation (F1-score = 0.68), the model performed comparably or even slightly better than a human annotator for segmenting MPs. However, over-sensitivity of the model can be suspected as the automatic model detected particularly high MPs levels across the three islands studied.

#### 4.1.2. Blooming artifact mitigation and mass estimation accuracy.

Blooming artifacts represent a previously under-recognized source of error in NR-based MP quantification. While these artifacts do not strongly affect particle counts, very large MPs may fragment into multiple detected regions. Mass estimates, calculated from projected areas, are more sensitive: overexposed particles artificially inflate apparent particle area, leading to slight mass overestimation. Our multi-spectra composite approach mitigates this effect by combining channels with different exposure characteristics. The differential exposure between our DAPI (typically overexposed) and TRI (lower exposure) channels allows the composite to constrain particle boundaries more accurately than single-channel imaging. Examination of the MP-SET dataset [20] suggests that blooming may contribute to the ~ 107% recovery rate reported for spiked samples – slight overestimation of particle areas would lead to mass overestimation even when particle counts are accurate. The 107% recovery rate for MP-NET on spiked samples may reflect blooming-induced area overestimation. While particle counts were accurate (detection was successful), the slight mass overestimation suggests systematic inflation of particle boundaries in single-wavelength fluorescence imaging. Several factors support this interpretation: (1) mass calculations from the projected area are sensitive to even small boundary errors (area scales as radius^2^); (2) spiked virgin MPs likely exhibited strong uniform fluorescence, maximizing blooming potential; and (3) recovery was calculated by mass, not by particle count.

Our composite approach, validated against µFTIR on real environmental samples (which exhibit more variable fluorescence and weathering), provides a framework for addressing both count and mass calibration in NR-based workflows. When combined with µFTIR validation (Section 4.2), this provides a calibration pathway for correcting systematic biases in mass estimates. The differential exposure strategy inherently constrains blooming artifacts while maintaining sensitivity across diverse polymer types, making it particularly suitable for complex environmental matrices where particle fluorescence intensity varies widely.

### 4.2. Comparing NR and µFTIR for accurate assessment of MPs contamination

#### 4.2.1. NR overestimation and general methodological challenge.

Since the NR staining method can lead to overestimates depending on the composition of the marked samples [52], a comparison of levels quantified by NR with those quantified by µFTIR was carried out for the island of Tubuai. These results show an overestimation by the NR staining method, due to the composition of our samples which leads to false positives [17,18]. The absence of correlation between NR particle counts and µFTIR-confirmed polymers further supports this interpretation. Because NR stains hydrophobic materials indiscriminately, fluorescence signals may partly originate from biological residues rather than plastics; this explains why no proportional relationship is observed between NR counts and polymer abundance. The removal of natural organic matter from samples has become a major challenge in NR fluorescence imaging [17] and is a crucial step for its use. Thus, digestion and extraction methods must be adapted to the natural organic load of the samples within the same matrix or across different matrices (i.e., aquatic, biological, sedimentary), depending on the non-plastics compounds found (see following section).

#### 4.2.2. Sample composition and digestion protocol.

The large mass and complex composition of giant clam tissues required careful digestion. For context, these specimens were considerably heavier (up to 123.1 ± 7.6 g for specimens from Tubuai, S1 Table) than typical bivalve bioindicators (e.g., an average weight of 20 g for *Mytilus spp* or 80 g for *Crassostreas gigas* [53]). A two-step protocol was therefore applied to viscera, including a relatively low concentration of nitric acid (20%), selected to balance tissue digestion efficiency and MP preservation, following the protocol of Schirinzi et al. [32]. In their study, some alterations of certain polymers (e.g., PA and PET) have been reported under low organic load conditions, when nitric acid was applied at the filtration stage after an initial KOH digestion at 60°C; although polymer mass and FTIR-based identification were not affected [32]. Applying the same protocol in *T. maxima* at a reduced KOH temperature (40 °C) produced similar outcomes, with all polymers fully recovered and no significant changes in particle length or width. FTIR spectra of PA and PE displayed minor new peaks, consistent with subtle chemical modifications rather than extensive degradation. By comparison, another study on giant clams [10] used a more aggressive protocol to digest the digestive system and gills, in order to analyze manufactured PE microbeads. In their *ex-situ* study, use of 70% HNO_3_ for 12 hours at 90°C resulted in a slight decrease in microbead fluorescence. Such harsh conditions may be acceptable for controlled experiments using virgin polymers, but are not suitable for environmental samples, as weathered MPs can be degraded at these concentrations and temperatures [50]. According to the proportion of proteins and stearate in our sample, further methodological developments are needed to improve flesh digestion of the organism without damaging MPs.

Among currently available approaches, enzymatic digestion has proven particularly effective [54], though its high cost limits its application in resource-limited contexts such as SIDS. Coupling digestion with density separation is generally not recommended, as the density of proteins (1.05–1.40 g cm^−3^) overlaps with that of common marine plastics (0.85–1.58 g cm^−3^) [55]. Increasing acid concentration could improve tissue removal, as gills, which did not undergo nitric acid digestion, showed higher rates of NR overestimation compared to viscera, treated with nitric acid. Any such adjustments must be carefully evaluated to avoid compromising polymer integrity, as subtle impacts have been noted for PE and PA in our study. Finally, although a sequential KOH–H_2_O_2_ oxidative digestion was not sufficient in our tests, the use of Fe^2^⁺ as a catalyst in Fenton’s reagent may represent a more efficient alternative. This process generates highly reactive radicals, enhancing organic matter degradation and outperforming conventional H_2_O_2_ oxidation, particularly in complex biological matrices such as benthic macroinvertebrates and other organic-rich samples [56,57]. Importantly, this approach has also been reported to effectively reduce fluorescence background from natural organic matter while preserving NR-stained MPs, thereby mitigating quenching and blooming artifacts in fluorescence-based detection workflows [17,58]. The efficiency of these digestion methods should be further investigated, particularly with regard to fibers (especially in the viscera) and certain particle size classes (i.e., 20–50 µm, 50–100 µm), as they currently appear to be responsible for the overestimation of NR compared with µFTIR. Indeed, DAPI filters have been reported to highlight non-plastic fibers, such as cellulose, linen, and cotton [17,18,59]. Tissue composition may also contribute to the variability recorded in NR fluorescence signals. In particular, lipid-rich matrices identified by µFTIR (especially in viscera) may promote non-specific hydrophobic interactions with Nile Red, increasing background fluorescence and affecting organ-to-organ comparisons [16–18]. This should be considered as a potential confounding factor in NR-based inter-organ comparisons. These findings highlight that both sample composition and detection parameters can influence quantification accuracy, emphasizing the need for careful calibration of the NR model and optimization of the fluorescence detection setup.

#### 4.2.3. Model calibration and optical setup.

Beyond sample preparation, the NR model itself can influence quantification accuracy, particularly through its inadequate sensitivity which may generate “false positives”. To manage this parameter, it would be advisable to adjust the model output score, which is based on the relative contrast of the MP previously defined, under the assumption that a plastic particle fluoresces more than a non-plastic particle. For the island of Tubuai, the use of scores of 0.20 for viscera and 0.26 for the gills give results both very close to those of µFTIR (0.61 ± 0.11 particles.g^-1^ ww and 2.86 ± 0.66 particles.g^-1^ ww respectively), and with similar trends for the two methods for all the stations studied. This demonstrates that appropriate score can mitigate overestimation. However, before applying new sensitive scores to models, additional µFTIR analysis from islands other than Tubuai are required to confirm that these score thresholds are broadly applicable, and that overestimation is not site-specific. Furthermore, the choice of fluorescence filters warrants investigation. For samples with low organic matter (OM) content, FITC and TRITC filters are appropriate, enabling the detection of a wide range of polymers (including PE, weathered HDPE, PET, PP, PVC, PS, EPS, and CA). However, in samples containing OM residues, these filters can produce numerous false positives [17,18]. In such cases, the use of UV wavelengths (e.g., 254 nm, 365 nm) or blue wavelengths (e.g., DAPI filter) is recommended [17]. Nevertheless, even these filters have limitations, as they may still detect non-plastic particles (i.e., cellulose, linen and cotton fibers), while failing to identify certain plastic polymers (i.e., altered PE, virgin HDPE, PS, PVC, nylon), which can result in an underestimation of plastic contamination [17,18,59]. Some studies have also conducted counter-labeling of OM using water-based hydrophilic dyes (such as methylene blue, Calcofluor white, Evans blue, and DAPI) after NR staining to differentiate OM particles from MPs [17]. In cases where co-staining dyes are used, only one blue fluorescence channel is employed due to the complexity of thresholding different channel colors. However, research is ongoing to improve the distinction between these thresholding techniques and enable differentiation between OM and MPs [17]. Such investigations on intercalibration and OM reduction will constitute the next step of our work.

#### 4.2.4. Perspectives and advantages of NR.

These investigations are crucial to improve NR methodology and our model. NR is an alternative that does indeed offer advantages in sample processing time and cost-effectiveness, applicable in resource-limited areas. It is a relatively straightforward method to implement and apply, allowing for the processing of a large number of samples. Recently, the method has successfully distinguished plastic polymers of different polarities (PA, PE, PET, PP, PS, PUR, PVC) using NR according to RGB colors with an accuracy of 80%, but it has not yet been applied to weathered samples and environmental samples [60]. However, there are still perspectives regarding the development of a standardized, reproducible method for the degradation of natural organic materials depending on the matrices considered, as well as for the NR detection method. Various factors can influence polymer detection and need to be standardized (e.g., choice of solvent, excitation wavelength, image capture and analysis, false positives related to natural organic matter, fluorescence variability due to polymer degradation in the environment, etc.) [17]. Regarding the solvent used, the protocol applied in this study (acetone/ethanol, 0.5 mL of a 20 µg·mL^−1^ solution at 50 °C for 10 min) follows Konde et al. [33], who optimized solvent type, concentration, temperature, and incubation time. However, recent work by Ho and Masura [58] suggests that acetone/water mixtures under controlled staining conditions (10 μg·mL^−1^ NR, 30 min, 70 °C), may improve fluorescence consistency across polymers, highlighting solvent composition as an additional parameter for standardization in NR-based workflows. These findings indicate that solvent selection may influence NR staining performance and deserves further investigation in environmental applications. In addition, post-staining chemical treatments such as sodium hypochlorite (14%) have been proposed to further reduce background fluorescence from residual organic matter on filter substrates, thereby improving image interpretability in fluorescence-based workflows [17]. However, such approaches remain application-dependent and require further validation across different sample matrices and imaging conditions.

#### 4.2.5. µFTIR limitations.

It is also important to consider the possible limitations of the µFTIR method, notably linked to the misidentification of weathered plastics polymers. It is recognized that environmental weathering induces a modification of the physical-chemical properties of MPs (i.e., crystallinity, mechanical properties and oxygen-containing groups) [61], which can alter the spectral signature of weathered plastic polymers compared to their virgin counterparts [62,63]. The use of a spectral library that reflects the compounds analyzed in environmental samples is thus of paramount importance. It is therefore advisable to use databases that include a large number of spectra of different non-plastic and plastic polymers that exhibit different weathering states (e.g., reference spectra from MPHunter, FLOPP and FLOPP-e), which improve the accuracy of compound identification [61,64]. In this study, despite employing a comprehensive spectral library (MPHunter), up to 11.3% of particles analyzed by µFTIR in viscera could not be identified. While further research could help clarify the identity of these particles, this relatively low proportion indicates that µFTIR provides nevertheless a reliable estimation of contamination, and this limitation appears to play a minor role in the discrepancies observed between NR and µFTIR.

#### 4.2.6. Environmental contamination levels and sources.

The high levels of MPs contamination observed through NR analyses across all three islands studied were also confirmed by µFTIR analysis, notably at 0.80 ± 0.16 particles.g^-1^ ww (at pool scale) in Tubuai. This contamination level was comparable to much more heavily anthropized regions, such as the oyster *Magallana bilineata* in India (0.81 ± 0.03 particles.g^-1^ ww [65]) and the clam *R. philippinarum* in China (0.78 ± 1.10 MP.g^-1^ ww [66]). However, these levels remained well below those previously reported in FP, where µFTIR analyses revealed higher contamination of *Pinctada margaritifera* collected from three pearl-producing atolls, reaching up to 7.3 ± 1.1 particles.g^-1^ ww, likely linked to pearl farming activities [4].

To further contextualize the contamination patterns observed across the islands, complementary µFTIR analyses on Tubuai samples illustrate potential local sources beyond pearl farming, which is absent on this island. The detected polymers suggest contributions from local agriculture, such as PVC (used in irrigation pipes and greenhouse sheets, 12.6% at pool scale) and PP (agricultural twines, 6.3%) [67], as well as maritime activities, including PA (fishing lines, 28.9%), PVC (floats), PS (10.8%) and PP (fishing nets)[68–70]. Waste mismanagement also appears important, with domestic refuse and food packaging that could contribute through polymers such as PET (*i.e.,* used in bottles, 10.6%), PS (*i.e.,* trays), and PA (*i.e.,* food film) [71]. The giant clam thus proved useful in highlighting potential local sources, similar to observations in China where *T. crocea* contamination was linked to packaging on Hainan island (with a predominance of Cellophane (70%), PA (9%) and PET (8%) [12]). Beyond local sources, MPs contamination on Tubuai may also originate from offshore and global inputs. Located near the South Pacific gyre, French Polynesia is exposed to floating plastics carried by ocean currents [5], with previous reports showing high MP accumulation on remote islands (e.g., Henderson Island: 3333 ± 1973 MP·kg^−1^ dw [68]). Regular lagoon–ocean exchanges may bring these distant plastics into Tubuai, meaning that giant clams likely integrate both local and global contributions to MPs contamination.

Such widespread exposure underscores the importance of developing robust, low-cost, and scalable detection methods to monitor MPs, particularly in remote or resource-limited regions such as SIDS. NR-based approaches, combined with machine learning, hold the potential to provide rapid quantification and spatial comparisons, representing a high-throughput alternative when extensive µFTIR analyses are impractical. Overall, the primary contributions of this study remain methodological, providing a framework for improving sample preparation, fluorescence detection, and machine learning–based automatic quantification in complex biotic matrices.

## 5. Conclusion

This study is the first to propose a methodological development of MPs quantification within the giant clam, developing ways of extracting MPs from this complex matrix, and using an original quantitative approach based on the Nile Red detection method. The automatic quantification model developed is innovative in its machine learning approach, learning from a multitude of photo profiles and based on prediction processes. This type of tools should play an increasing role in the field of MPs pollution, differing from current quantification tools in dealing with complex images derived from several fluorescence channels, with the aim of maximizing the detection of plastic polymers. However, there is still room for improvement, given the overestimation of the levels quantified by the NR method, compared with µFTIR spectroscopy, in terms of defining the fluorescence sensitivity threshold of the model (referred here as the “score”), but also in terms of improved digestion methods for extracting MP. This last point demonstrates the limitations of current purification methods for biota in the literature, applied to giant clams. Due to its ease of use and low cost, and despite its drawbacks, NR-based analysis will continue to play a major role in monitoring MPs in island areas, particularly those with low or intermediate economic levels (e.g., SIDS). Continuing to refine NR methodologies and machine learning approaches, such as those developed in this study, constitutes a promising frontier for MP research. By integrating advanced image recognition, adaptive learning algorithms, and standardized reference datasets, such tools could ultimately provide rapid, reliable, and scalable monitoring of plastics contamination across ecosystems.

## Supporting information

S1 Table Shell length and tissue wet weights for individuals *T. maxima* specimens analyzed by NR fluorescence microscopy or µFTIR. (PDF)

S2 Table Data augmentation configuration. (PDF)

S3 Table Optimizer configuration. (PDF)

S4 Table Correlations between NR- and µFTIR-detected particle concentrations across organs (gill, viscera, pooled) of *T. maxima* for total abundance, shape and size. Data show Spearman’s rho correlation coefficients and associated p-value (n = 13).
(PDF)

S1 Fig Mean (± SE) wet masses of dissected gills and viscera of *T. maxima* per island (n = 15 per island). Bars show the average masses derived from the NR dataset for Makemo, Hao, and Tubuai; error bars denote standard error.
(TIF)

S2 Fig Data preparation pipeline of the in-house dataset (13402 raw images). Composite images are generated through a three-stage process (see Section 2.3.1). Selected images were annotated semi-automatically with Labkit through batch annotation tasks, while the remaining images were annotated automatically using model predictions. The resulting masks were compiled into the FiftyOne framework, which enabled cluster-based analysis to identify poorly annotated samples and create correction tasks. This pipeline facilitated efficient processing of large image volumes while ensuring high-quality annotations for machine learning-based MPs quantification. Those composite images were then sampled for semi-automatic labeling in order to create a training and validation set for modeling experiments. Unlabeled data were automatically labeled using the final model once modeling experiments were conclusive.
(TIF)

S3 Fig U-Net architecture used for particle segmentation. (TIF)

S4 Fig Images of five different polymers (N = 3) incorporated into giant clam viscera, shown before digestion (CTRL), after the first digestion step (10% KOH at 40 °C for 48 h), and after the second digestion step (10% KOH at 40 °C for 48 h followed by 20% HNO_3_ at room temperature for 1 h). The polymers examined are polypropylene (PP), polyethylene (PE), polyvinyl chloride (PVC), polyamide (PA), and a PE/PP copolymer (pearl-farming rope).
(TIF)

S5 Fig Variation in polymer width (mean ± SE) across digestion steps. Width of polymer fragments (n = 3 per polymer type) measured before digestion (CTRL), after the first digestion step (10% KOH, 40 °C, 48 h), and after the second digestion step (10% KOH followed by 20% HNO_3_). Different letters indicate significant differences among digestion steps (PERMANOVA, p < 0.05).
(TIF)

S6 Fig Variation in polymer length (mean ± SE) across digestion steps. Length of polymer fragments (n = 3 per polymer type) measured before digestion (CTRL), after the first digestion step (10% KOH, 40 °C, 48 h), and after the second digestion step (10% KOH followed by 20% HNO_3_). Different letters indicate significant differences among digestion steps (PERMANOVA, p < 0.05).
(TIF)

S7 Fig FTIR spectra of the five polymers (PP, PE, PVC, PA, PE/PP copolymer) incorporated into giant clam viscera, recorded before digestion (CTRL) and following each digestion stage (first and second digestion). (PDF)

S8 Fig Side-by-side comparison of microplastic (MP) detection in two representative regions of interest (ROIs). Images are arranged in two columns, one per ROI. The top row shows the original fluorescence images, while the bottom row shows the corresponding annotations and predictions: human/Labkit annotations are highlighted in purple, and U-Net predictions are shown in gray.
(TIF)

S1 Protocol Annotation MP procedure with Labkit software. (PDF)
