# Supplementary material for: Exploring Nile Red and machine learning for microplastics detection in Tridacna maxima
Source: PLoS One. 2026 Sep 16;21(9):e0357014. doi: 10.1371/journal.pone.0357014 (PMC13581202; doi:10.1371/journal.pone.0357014)
Supplement: Supplementary file 1 [file pone.0357014.s001.pdf]

Analysis	Sample	Shell length (mm)	Total weight (g, ww)	Gill weight (g, ww)	Viscera weight (g, ww)
NR	TB-S1-P1	180	161.04	6.62	49.76
NR	TB-S1-P2	153	119.33	3.69	40.15
NR	TB-S1-P3	154	134.53	4.86	63.67
NR	TB-S1-P4	168	182.10	6.42	74.44
NR	TB-S1-P5	173	175.01	6.06	70.37
NR	TB-S2-P1	147	105.91	3.96	29.70
NR	TB-S2-P2	149	90.86	4.02	29.40
NR	TB-S2-P3	146	95.02	3.42	32.73
NR	TB-S2-P4	172	106.61	3.55	35.00
NR	TB-S2-P5	142	100.63	3.68	31.03
NR	TB-S3-P1	147	101.82	3.75	38.14
NR	TB-S3-P2	154	108.53	4.52	40.98
NR	TB-S3-P3	165	124.85	4.54	51.21
NR	TB-S3-P4	162	101.75	4.00	38.12
NR	TB-S3-P5	182	138.60	5.90	62.25
NR	HAO-S1-P1	145	71.64	2.82	17.99
NR	HAO-S1-P2	140	84.37	3.40	21.31
NR	HAO-S1-P3	180	150.82	6.92	43.40
NR	HAO-S1-P4	160	93.75	3.46	28.34
NR	HAO-S1-P5	165	136.13	3.40	62.88
NR	HAO-S2-P1	160	99.56	3.47	25.02
NR	HAO-S2-P2	150	104.99	3.06	46.65
NR	HAO-S2-P3	150	99.37	3.37	35.42
NR	HAO-S2-P4	170	90.13	6.77	31.10
NR	HAO-S2-P5	165	116.32	4.69	29.97
NR	HAO-S3-P1	120	34.82	2.11	10.89
NR	HAO-S3-P2	135	64.06	2.55	31.46
NR	HAO-S3-P3	135	96.96	2.44	46.36
NR	HAO-S3-P4	145	59.15	3.20	18.57
NR	HAO-S3-P5	125	47.15	2.54	13.84
NR	MAK-S3-P1	129	103.47	4.29	29.03
NR	MAK-S3-P2	133	57.15	3.57	16.55
NR	MAK-S3-P3	170	31.06	2.39	7.52
NR	MAK-S3-P4	120	37.60	1.97	8.84
NR	MAK-S3-P5	133	63.11	2.57	15.88
NR	MAK-S2-P1	136	92.52	3.93	26.03
NR	MAK-S2-P2	146	108.95	3.92	41.03

NR	MAK-S2-P3	133	75.11	3.01	39.22
NR	MAK-S2-P4	131	59.61	2.98	25.35
NR	MAK-S2-P5	151	93.53	4.82	24.09
NR	MAK-S1-P1	145	65.58	2.81	16.49
NR	MAK-S1-P2	110	55.30	2.89	12.14
NR	MAK-S1-P3	109	124.03	4.52	47.15
NR	MAK-S1-P4	118	51.39	2.83	12.74
NR	MAK-S1-P5	123	48.19	3.11	16.98
µFTIR	TB-S1-P6	172	186.62	4.63	52.61
µFTIR	TB-S1-P7	190	113.80	5.17	55.91
µFTIR	TB-S2-P6	157	118.39	2.21	39.37
µFTIR	TB-S2-P7	175	132.27	3.94	55.25
µFTIR	TB-S2-P8	177	186.11	4.79	61.70
µFTIR	TB-S3-P6	175	149.18	5.19	44.82
µFTIR	TB-S3-P7	170	131.25	5.25	33.07
µFTIR	TB-S3-P8	178	149.05	4.89	59.33
µFTIR	TB-S3-P9	175	141.11	6.32	30.78
µFTIR	TB-S3-P10	178	155.43	5.66	62.84
µFTIR	TB-S3-P11	163	115.28	5.21	49.87
µFTIR	TB-S3-P12	165	120.35	2.79	40.43
µFTIR	TB-S3-P13	167	125.95	5.04	42.08
