# Supplementary material for: Exploring Nile Red and machine learning for microplastics detection in Tridacna maxima
Source: PLoS One. 2026 Sep 16;21(9):e0357014. doi: 10.1371/journal.pone.0357014 (PMC13581202; doi:10.1371/journal.pone.0357014)
Supplement: Supplementary file 2 [file pone.0357014.s002.pdf]

**Augmentation transform**	**Parameters**
Random Resize	scale= (625, 1000), ratio_range= (0.8, 1.2), keep_ratio= True)
Random Crop Foreground	crop_size= (400,400), cat_max_ratio= 0.75
Random Flip	
PhotoMetric Distortion	brightness_delta= 32, contrast_range= (0.7, 1.3), saturation_range= (0.7, 1.3), hue_delta=2
