# Supplementary material for: Exploring Nile Red and machine learning for microplastics detection in Tridacna maxima
Source: PLoS One. 2026 Sep 16;21(9):e0357014. doi: 10.1371/journal.pone.0357014 (PMC13581202; doi:10.1371/journal.pone.0357014)
Supplement: Supplementary file 3 [file pone.0357014.s003.pdf]

Optimizer	Parameters
SGD	lr= 0.01, momentum= 0.9, weight_decay= 0.0005, batch_size= 4
