# Supplementary material for: Exploring Nile Red and machine learning for microplastics detection in Tridacna maxima
Source: PLoS One. 2026 Sep 16;21(9):e0357014. doi: 10.1371/journal.pone.0357014 (PMC13581202; doi:10.1371/journal.pone.0357014)
Supplement: Supplementary file 4 [file pone.0357014.s004.pdf]

Organ	Sperman correla-tion	Total	Shape		Size range					
			Fiber	Fragment	25-50	50-100	100-200	200-500	500-1000	1000
Gill	roh	-0.214	-0.022	-0.099	-	0.286	-0.371	-0.060	0.523	-0.551
	p-value	0.482	0.949	0.751	-	0.343	0.213	0.849	0.066	0.099
Viscera	roh	-0.374	0.055	0.176	-	-0.162	-0.214	-0.324	0.181	0.176
	p-value	0.209	0.863	0.566	-	0.596	0.482	0.279	0.554	0.565
Pool	roh	-0.186	-0.434	-0.192	-	0.341	0.181	0.121	-0.049	0.407
	p-value	0.541	0.140	0.530	-	0.255	0.554	0.696	0.877	0.168
