# Supplementary material for: Exploring Nile Red and machine learning for microplastics detection in Tridacna maxima
Source: PLoS One. 2026 Sep 16;21(9):e0357014. doi: 10.1371/journal.pone.0357014 (PMC13581202; doi:10.1371/journal.pone.0357014)
Supplement: Supplementary file 5 [file pone.0357014.s011.pdf]

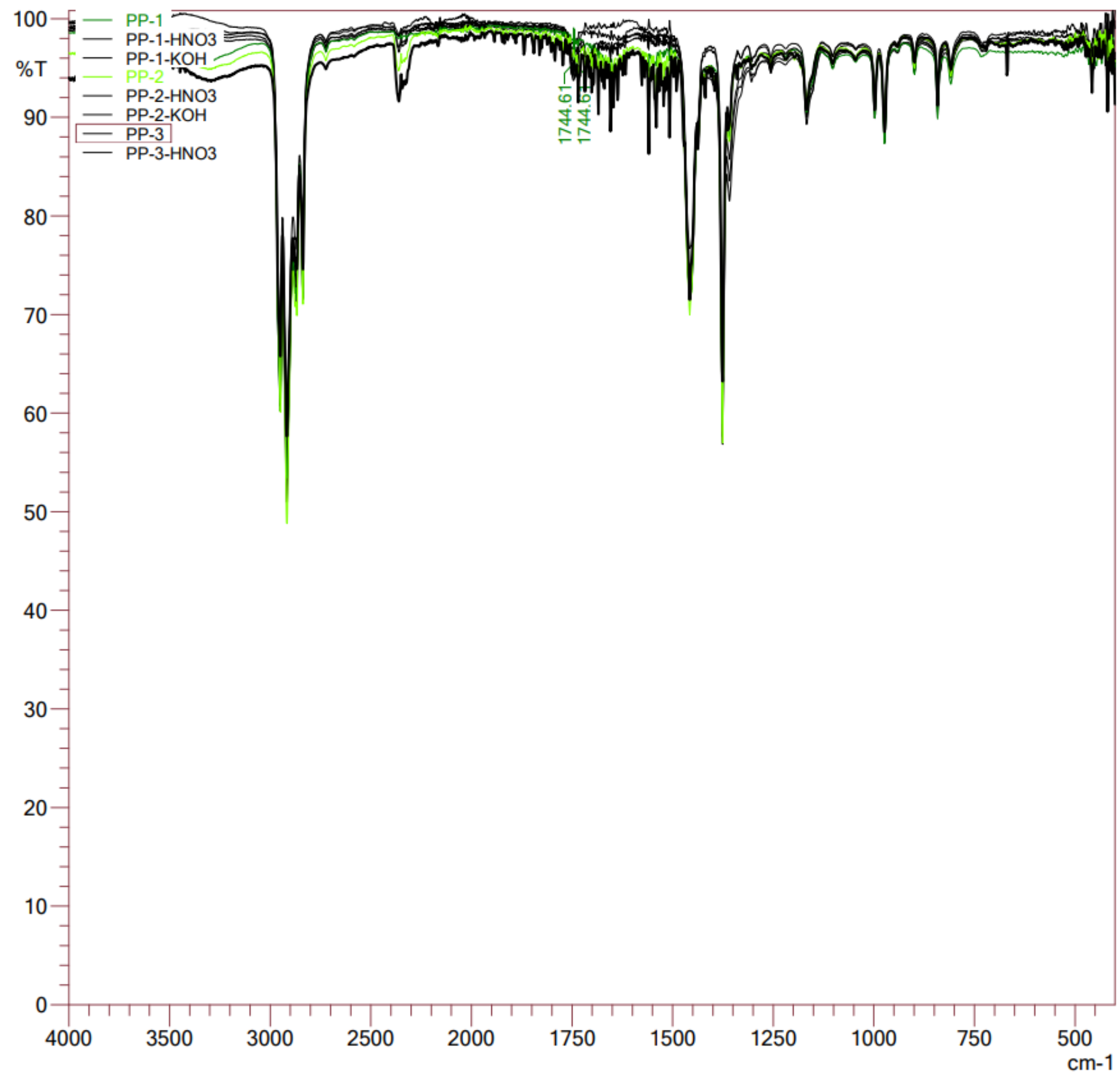

PP-1
PP-1-HNO3
PP-1-KOH
PP-2
PP-2-HNO3
PP-2-KOH
PP-3
PP-3-HNO3
%T
1744.61
1744.6
cm-1

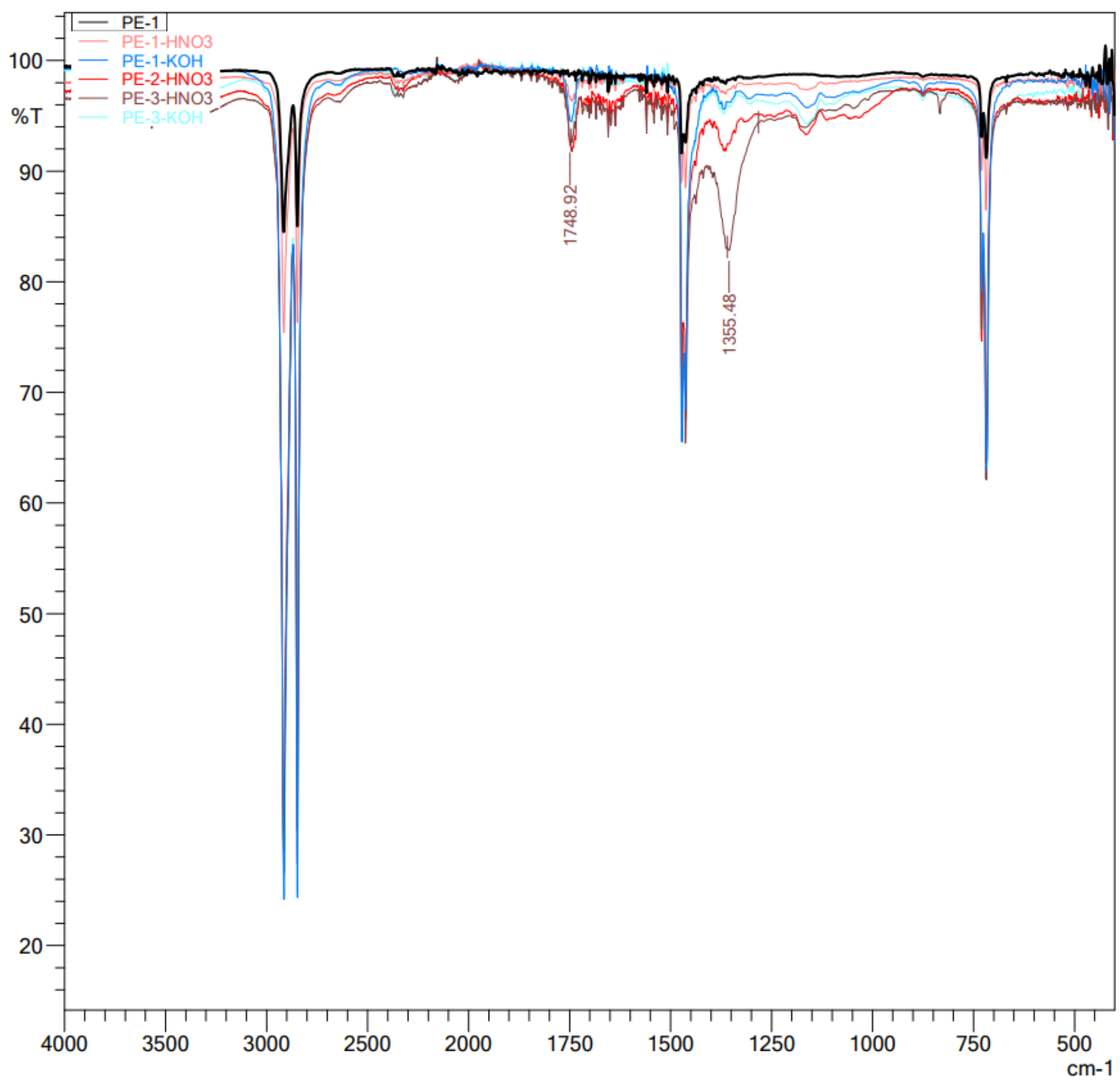

PE-1
PE-1-HNO3
PE-1-KOH
PE-2-HNO3
PE-3-HNO3
PE-3-KOH
%T
1748.92
1355.48
cm-1

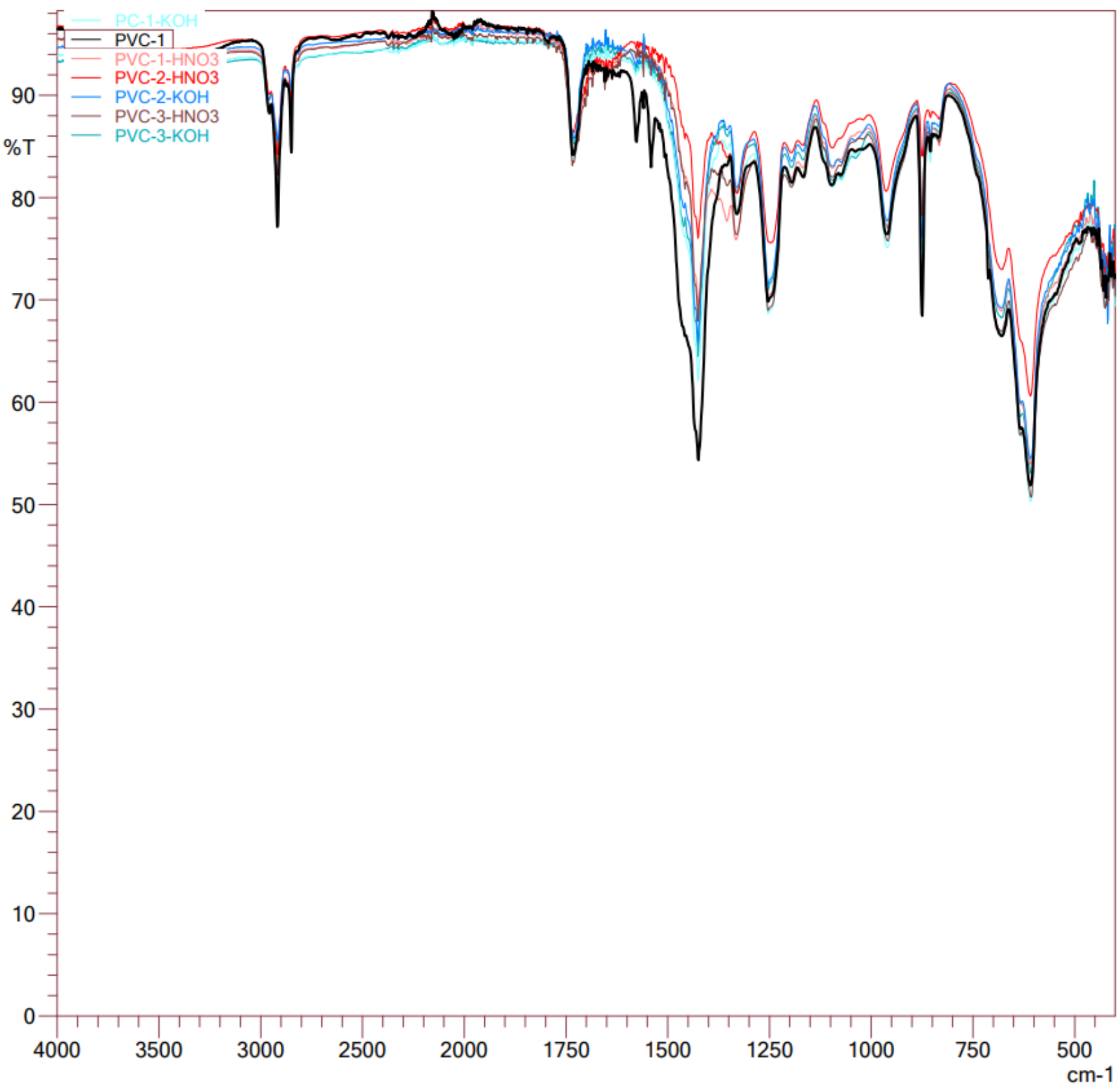
PC-1-KOH
PVC-1
PVC-1-HNO3
PVC-2-HNO3
PVC-2-KOH
PVC-3-HNO3
PVC-3-KOH
%T
0
10
20
30
40
50
60
70
80
90
4000
3500
3000
2500
2000
1750
1500
1250
1000
750
500
cm-1

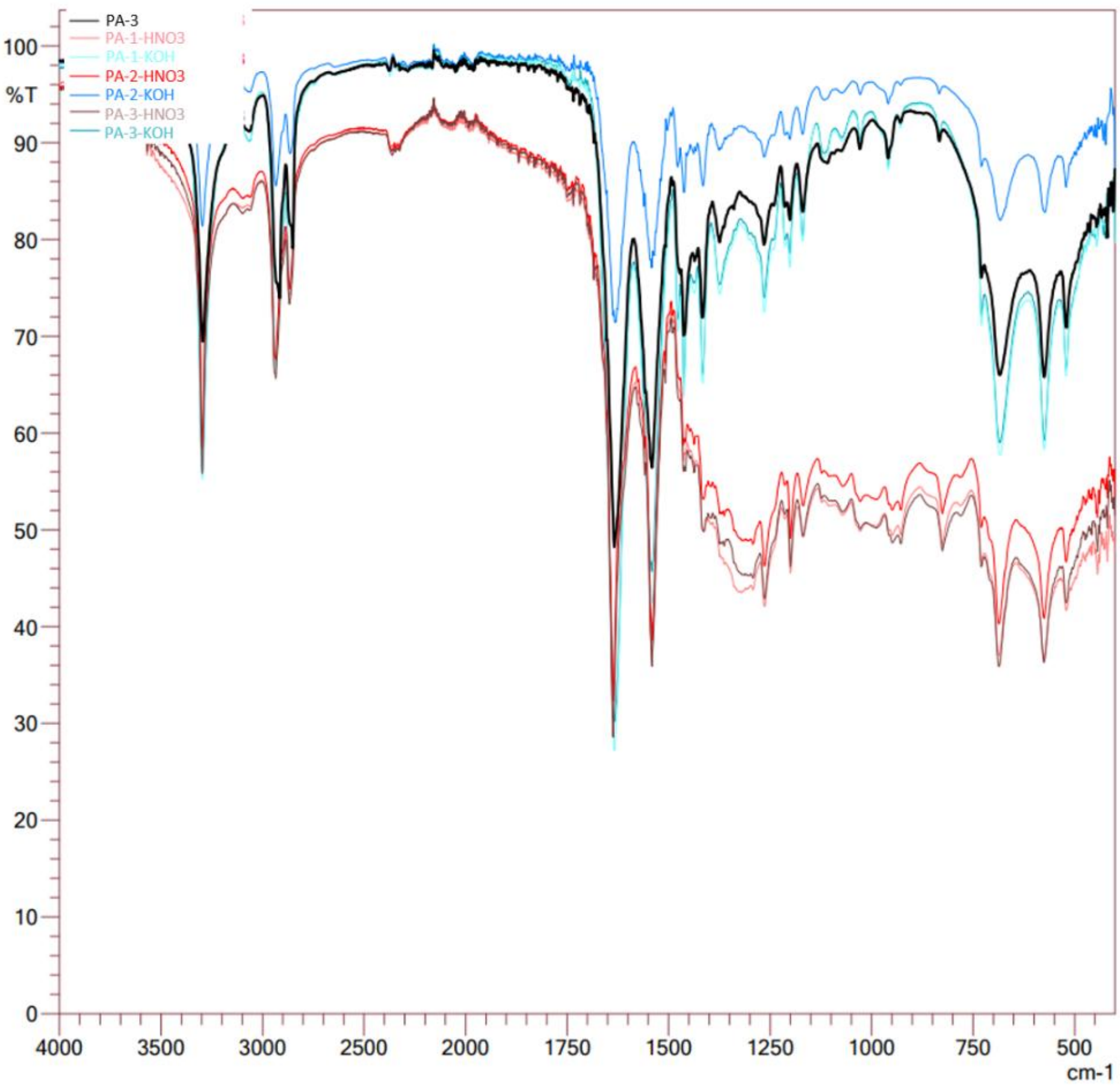
PA-3
PA-1-HNO3
PA-1-KOH
PA-2-HNO3
PA-2-KOH
PA-3-HNO3
PA-3-KOH
%T
0
10
20
30
40
50
60
70
80
90
100
4000
3500
3000
2500
2000
1750
1500
1250
1000
750
500
cm-1

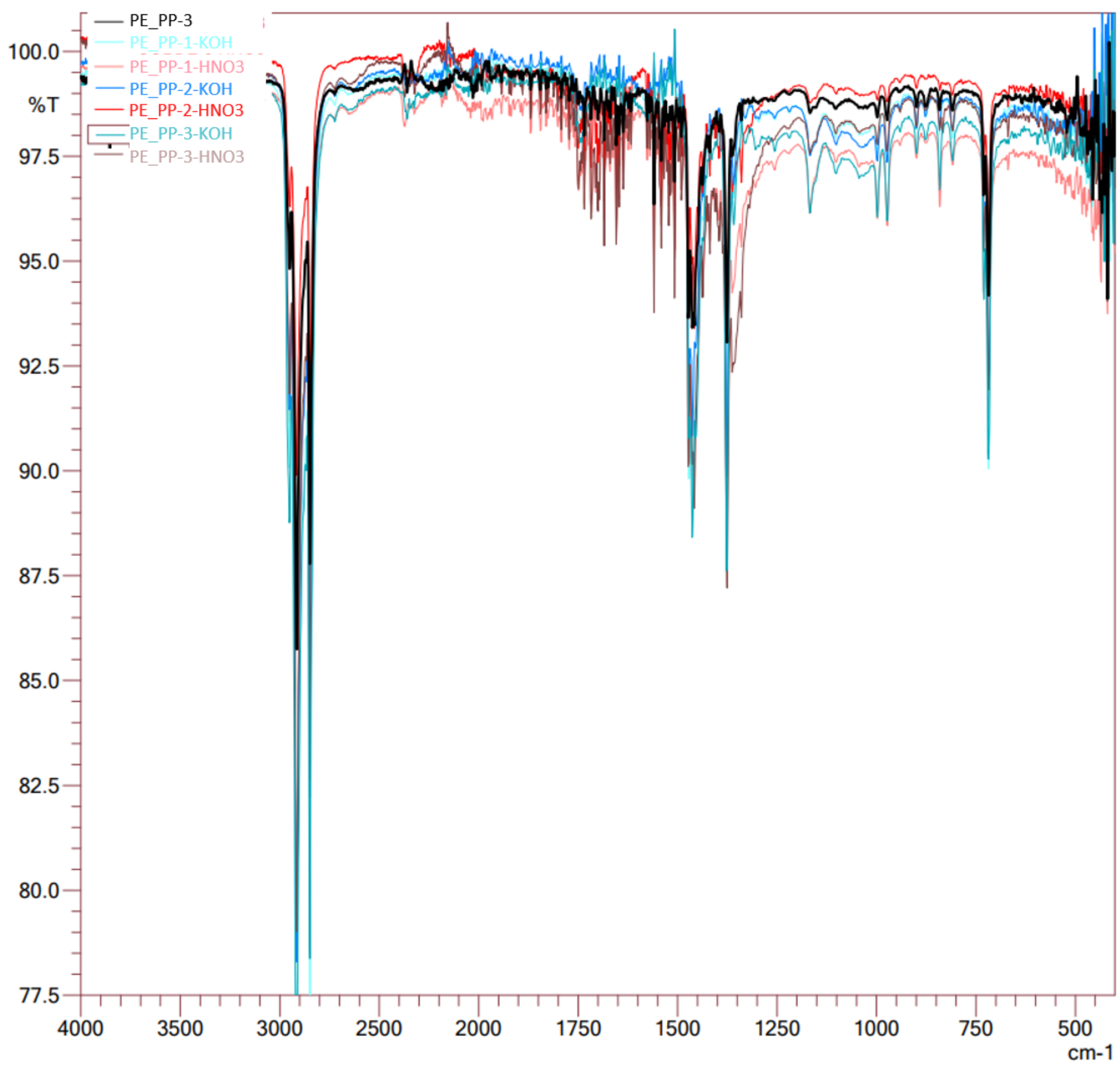
PE_PP-3
PE_PP-1-KOH
PE_PP-1-HNO3
PE_PP-2-KOH
PE_PP-2-HNO3
PE_PP-3-KOH
PE_PP-3-HNO3
%T
100.0
97.5
95.0
92.5
90.0
87.5
85.0
82.5
80.0
77.5
4000
3500
3000
2500
2000
1750
1500
1250
1000
750
500
cm-1
