# Supplementary material for: Exploring Nile Red and machine learning for microplastics detection in Tridacna maxima
Source: PLoS One. 2026 Sep 16;21(9):e0357014. doi: 10.1371/journal.pone.0357014 (PMC13581202; doi:10.1371/journal.pone.0357014)
Supplement: Supplementary file 6 [file pone.0357014.s013.pdf]

- Run Fiji
- Go to Import Image sequence, then select folder with images and set scale to 50%
- Go to Image, Properties and set: “slices (z)” to 1; “frames (t)” to number of frames; “frame interval” to 1
- Go to Plugin, Labkit, “Open current image with Labkit”
- From here, the Labkit procedure is divided into 7 steps:
  - Step 0: Run Labkit pixel classification → select GPU acceleration (if GPU available) and select basic default features. Experiments were conducted by adding features and changing the list of sigmas (parameters for Gaussian kernel descriptors). No significant improvement was observed thus default parameters are kept for the whole annotation process.
  - Step 1: Annotate the foreground pixels for three MPs and their background by delimiting their border with rough strokes as shown below (here, blue is used for the background, and red for the particles to be detected)

A

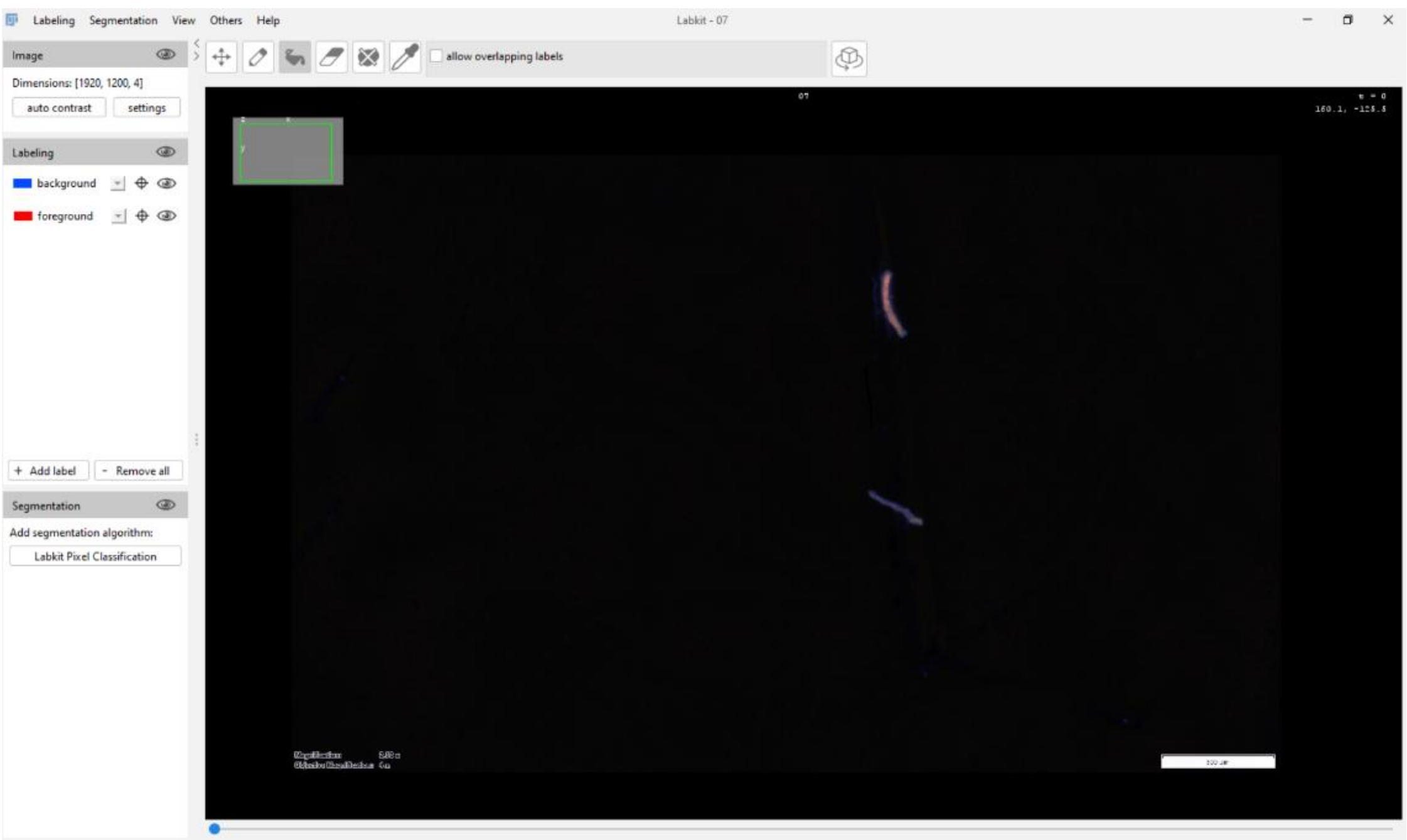

B

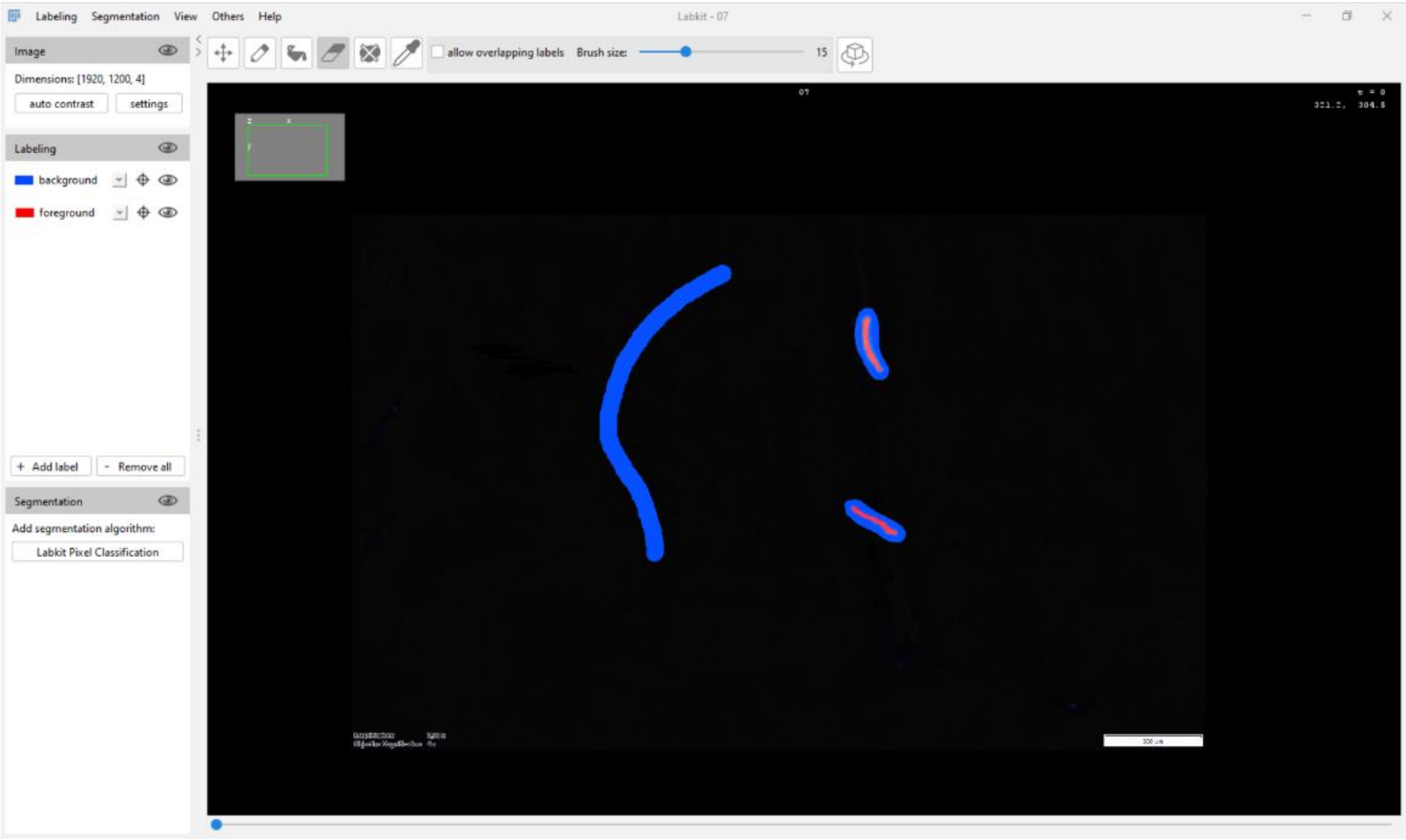

Fig A in protocol S1. Example of annotation procedure with Labkit in Fiji. (A) Original image showing fluorescent particles before annotation. (B) Annotated image showing foreground (red) and background (blue) labels used for pixel classification.

- Step 2: Train model (play button "Labkit Pixel Classification")
- Step 3: Fix invalid predictions by adding extra foreground/background strokes on invalid regions.
- Step 4: Repeat Step 2 and 3 until predictions on all batched images are perfect.
- Step 5: Save Labkit model.
- Step 6: use Fiji macro to perform inference and save annotation masks.
